# Supplementary material for: Structural basis of Fanconi anemia pathway activation by FANCM
Source: EMBO J. 2025 May 30;44(14):4013–36. doi: 10.1038/s44318-025-00468-3 (PMC12263834; doi:10.1038/s44318-025-00468-3)
Supplement: Supplementary file 1 — Appendix [file 44318_2025_468_MOESM1_ESM.pdf]

Appendix for “Structural basis of Fanconi Anemia pathway activation by FANCM”

Table of Contents

Appendix Figure S1: Supporting information for AlphaFold models 2
Appendix Figure S2: Predicted association point on FANCF/FANCC does not obstruct associations with the remainder of the FA core complex. 3

Appendix Table S1: Data collection and refinement statistics for crystal structures included in this manuscript 4
Appendix Table S2: Oligonucleotides used for the generation of nucleic acid substrates 5

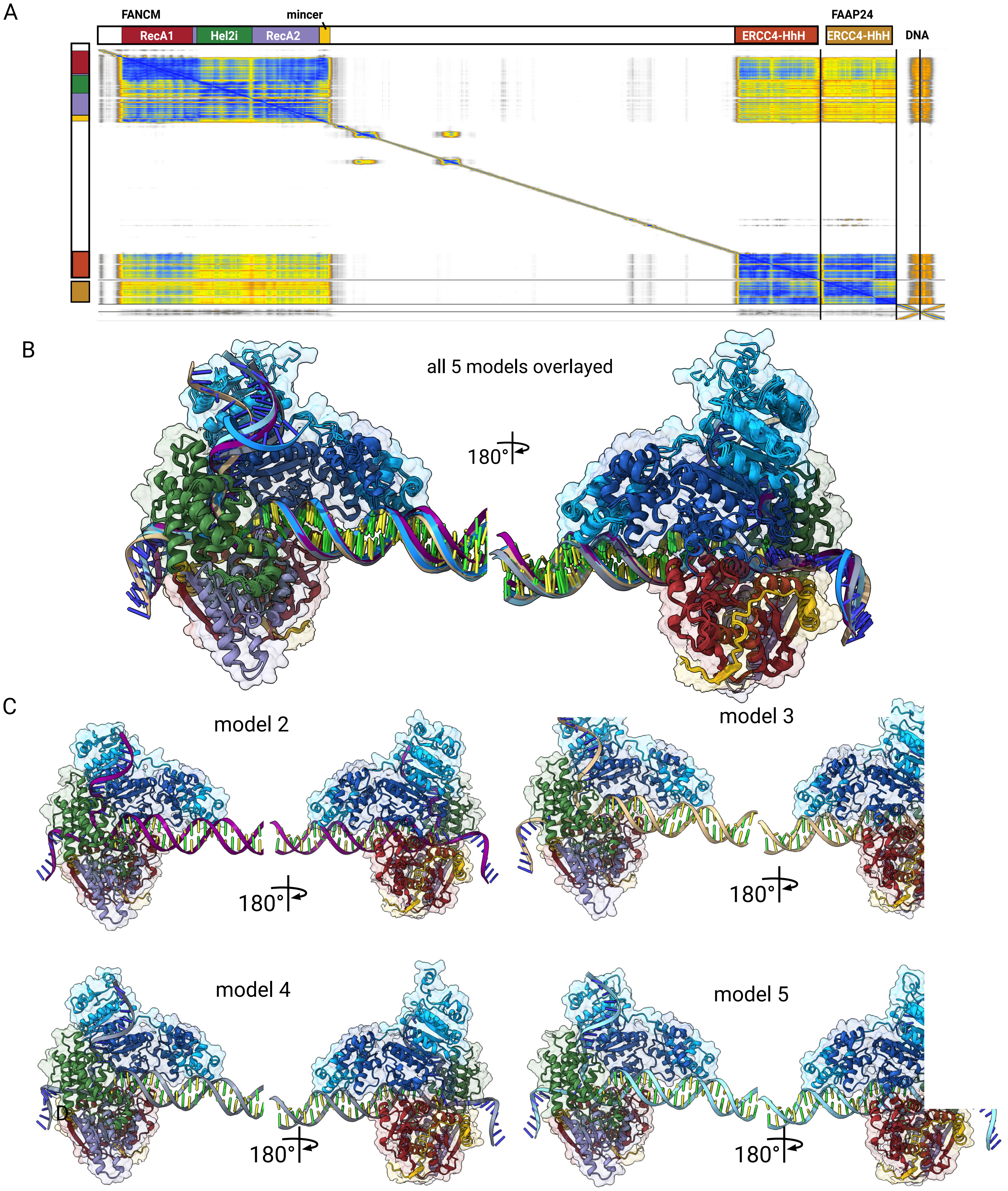

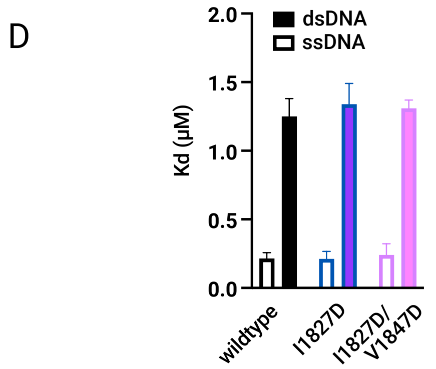

Appendix Figure S1. A) Predicted aligned error (PAE) plot for model shown in Figure 4A, with blue representing small errors (<5 Å), and yellow (10 Å), orange (15 Å), and gray (> 20 Å) representing larger estimated errors. B) All 5 AlphaFold models overlayed, and coloured according to Figure 4A. DNA is different colour in each model. C) Models 2-5 shown from 2 orientations (as in model 1 of Figure 4A). D) Relative DNA binding affinities of wildtype, I1827D and I1827D/V1847D FANCM-CTD:FAAP24 complexes are not altered.

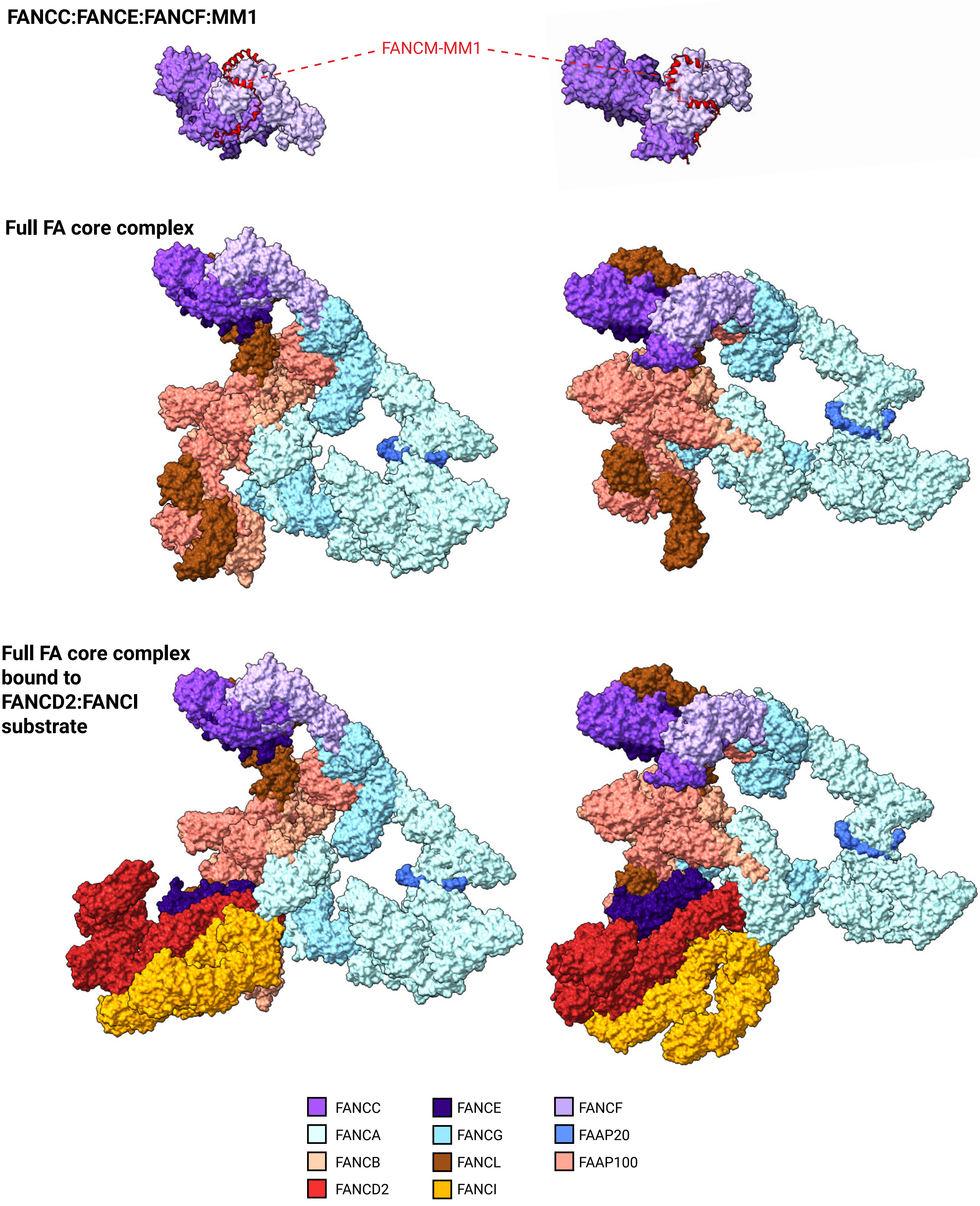

Appendix Figure S2: Predicted association point on FANCF/FANCC does not obstruct associations with the remainder of the FA core complex. Side-by-side AlphaFold model of FANCC/FANCE/FANCF/FANCM-MM1 model (top) and FANCC/FANCE/FANCF subcomplex within the full FA core complex (PDB:7KZP, middle) or FA core complex:FANCD2:FANCI substrate complex (PDB:7ZKQ, bottom).

Appendix Table S1: Data collection and refinement statistics for crystal structures included in this manuscript

	Translocase-DNA	ERCC4-like:FAAP24-DNA
**Data collection**		
Space group	P212121	P1
Cell dimensions		
a, *b*, *c* (Å)	80.94, 80.94, 93.39	61.01, 69.98, 86.77
α, β, γ (°)	90.00, 90.00, 90.00	91.63, 89.93, 115.95
Resolution (Å)	61.16-2.20 (2.27-2.20)	86.72-2.40 (2.44-2.40)
R_{merge}	0.08 (1.175)	0.092 (1.524)
R_{pim}	0.032 (0.473)	0.054 (0.928)
CC1/2	0.999 (0.844)	0.998 (0.344)
$I / \sigma I$	16.6 (2.2)	17.0 (1.1)
Completeness (%)	100.0 (100.0)	96.2 (70.2)
Redundancy	3.5 (3.7)	3.9 (3.7)
**Refinement**		
Resolution (Å)	2.20	2.40
No. reflections	33512	48487
R_{work} / R_{free}	0.22/0.27	0.21/0.26
No. atoms		
Protein	4345	6454
DNA	761	859
Water	80	114
B-factors		
Protein	57.35	66.91
DNA	76.91	108.03
Water	50.84	46.64
R.m.s. deviations		
Bond lengths (Å)	0.004	0.0068
Bond angles (°)	1.324	1.726

Appendix Table S2: Oligonucleotides used for the generation of nucleic acid substrates

Name	Sequence
Cy5/XOM1	/5Cy5/ACGCTGCCGAATTCTACCAGTGCCTTGCTAGGACATCTTTGCCCACCTGCAGGTTCACCC
XM3	GGGTGAACCTGCAGGTGGGCAAA**A**ATGTCCTAGCAAGGCACTGGTAGAATTCGGCAGCGT
Xam1/3Dab	GCCGAATTCTACCAGTGCCTTGCTAGGACATCTTTGCCCACCTGCAGGTT/3Dab/
Cy3/Xam2	/5Cy3/AACCTGCAGGTGGGCAAAGATGTCCATCTGTTGTAATCGTCAAGCTTTAT
Xam3	AACCTGCAGGTGGGCAAAAATGTCCTAGCAAGGCACTGGTAGAATTCGGC
Xam4	ATAAAGCTTGACGATTACAACAGATGGACATTTTTGCCCACCTGCAGGTT
Cy5/XOs1	/5Cy5/TACCAGTGCCTTGCTAGGACATCTTTGCCCAC
XOs2	GTGGGCAAAGATGTCCATCTGTTGTAATCGTC
XOs3	GACGATTACAACAGATCATGGAGCTGTCTAGA
XOs4	TCTAGACAGCTCCATGTAGCAAGGCACTGGTA
Hairpin15	GGTATGAGCACTGCTTAGGCAGTGCTCATACCGCATGGAGCTG
SA1	TACGCATCATCGCTCGGTTTT
SA2	TTTTCCGAGCGATGATGCGTA
