# Supplementary figures and images for: Structural basis of Fanconi anemia pathway activation by FANCM
Source: EMBO J. 2025 May 30;44(14):4013–36. doi: 10.1038/s44318-025-00468-3 (PMC12263834; doi:10.1038/s44318-025-00468-3)

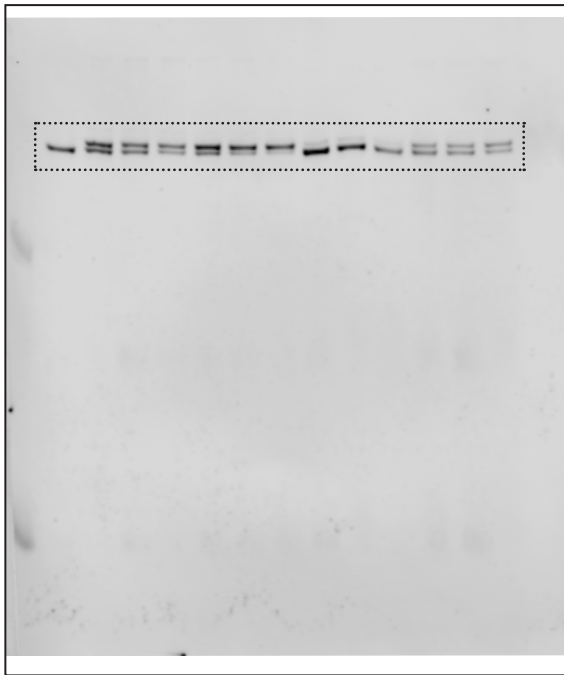

FANCD2

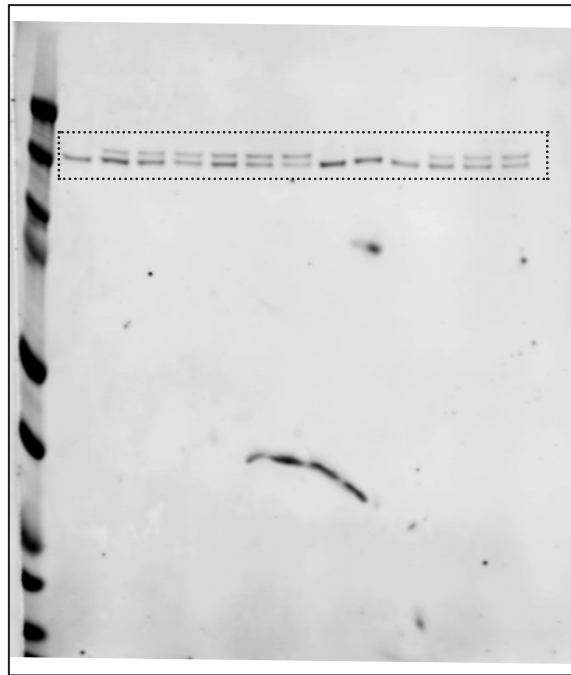

FANCI

repeat 1

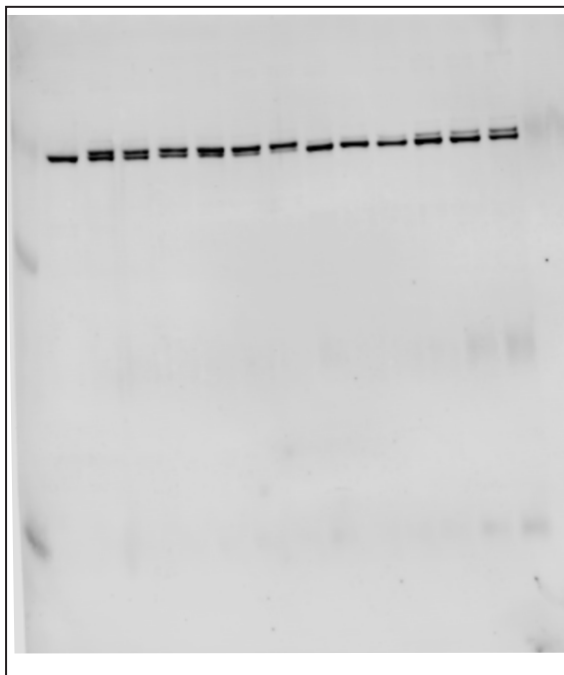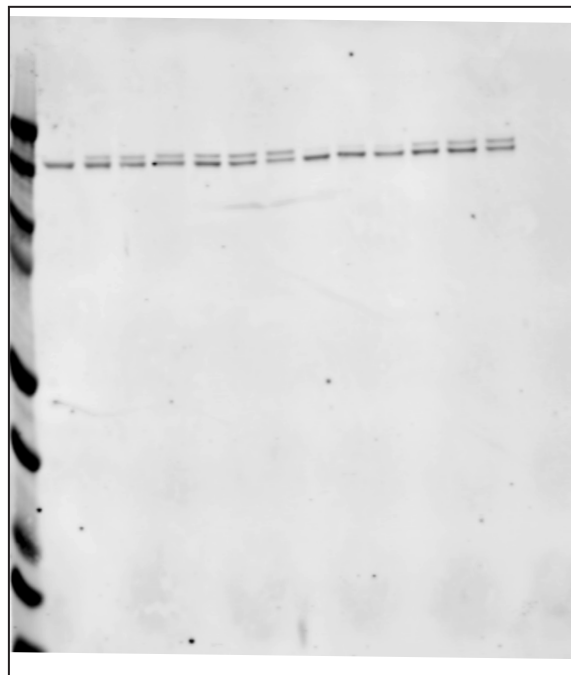

repeat 2

Supplement: Supplementary file 3 — Source data Fig. 1 [file 44318_2025_468_MOESM3_ESM.zip › Figure 1/1E/Uncropped data from Figure 1E.pdf]

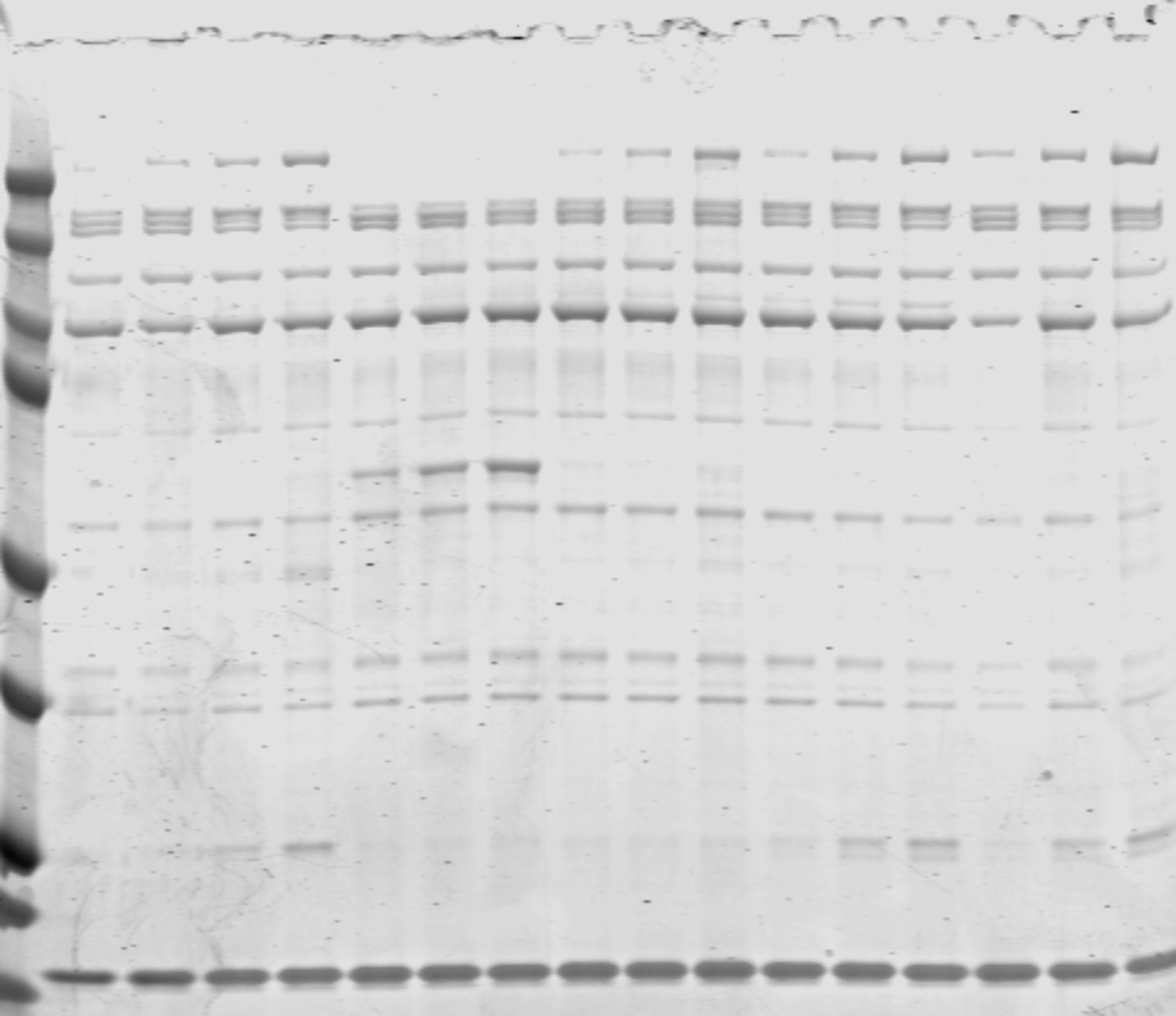

Supplement: Supplementary file 3 — Source data Fig. 1 [file 44318_2025_468_MOESM3_ESM.zip › Figure 1/1G/Coomassie gel uncropped.tif]

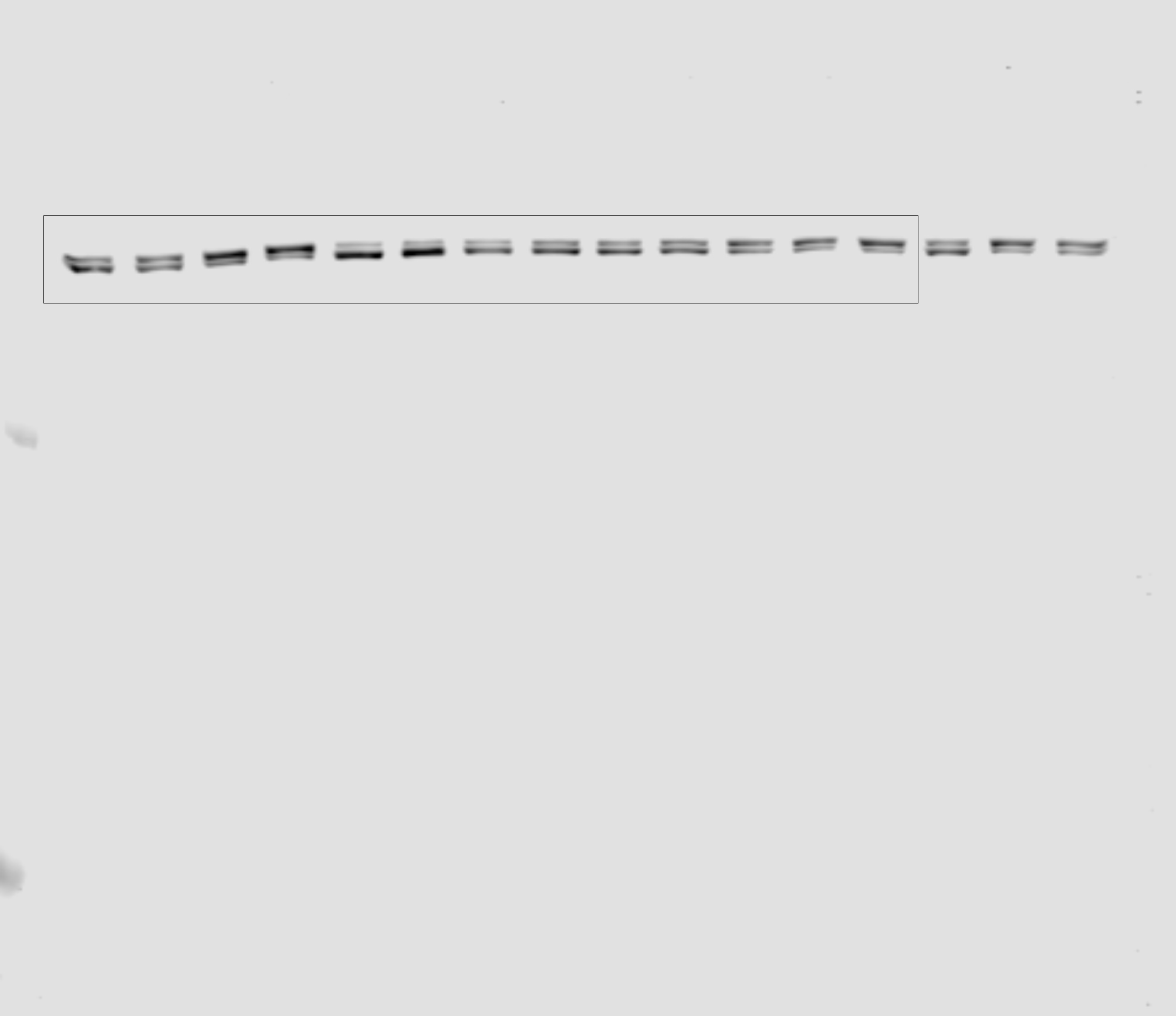

Supplement: Supplementary file 3 — Source data Fig. 1 [file 44318_2025_468_MOESM3_ESM.zip › Figure 1/1G/FANCD2 Western uncropped.tif]

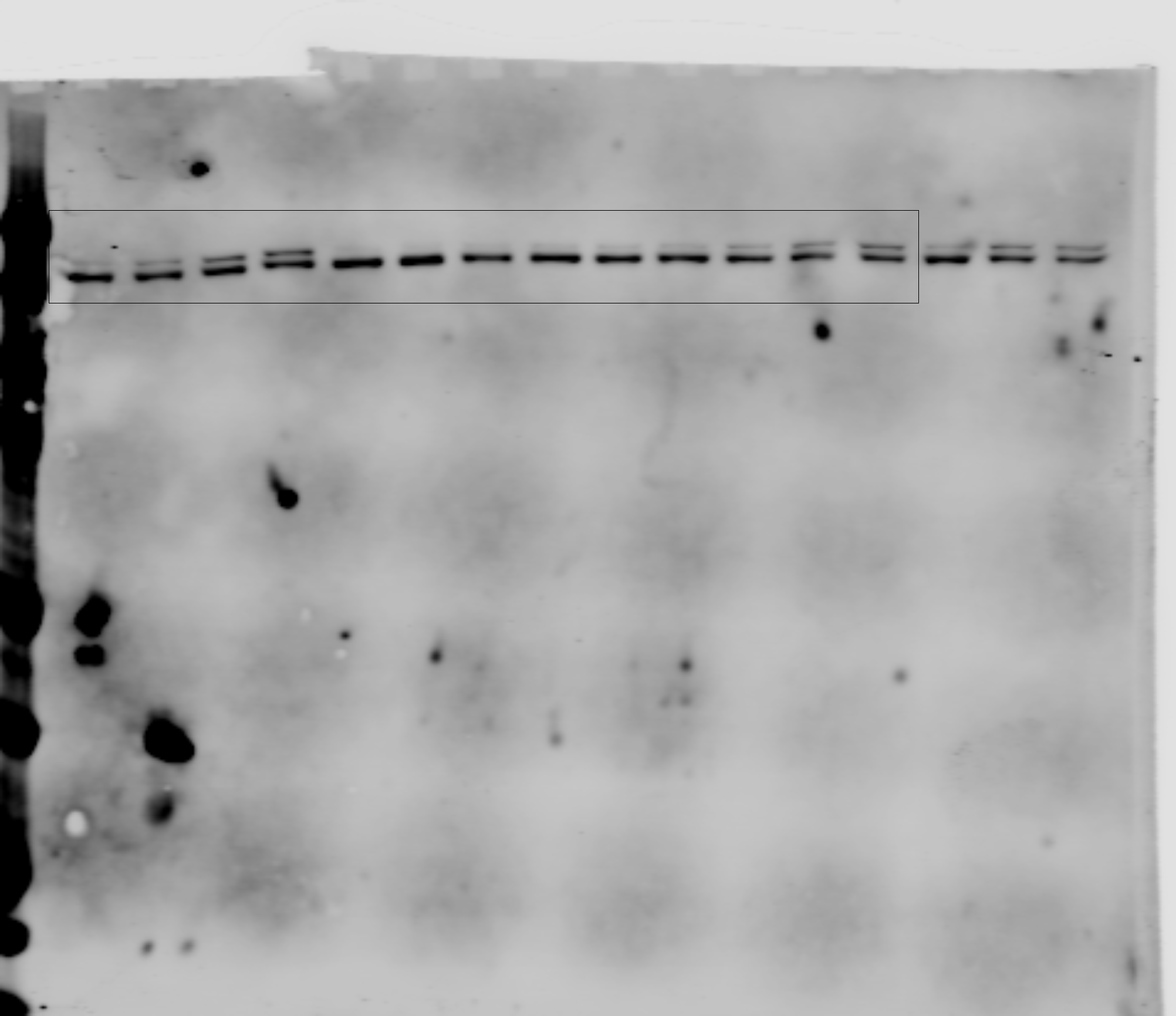

Supplement: Supplementary file 3 — Source data Fig. 1 [file 44318_2025_468_MOESM3_ESM.zip › Figure 1/1G/FANCI Western uncropped.tif]

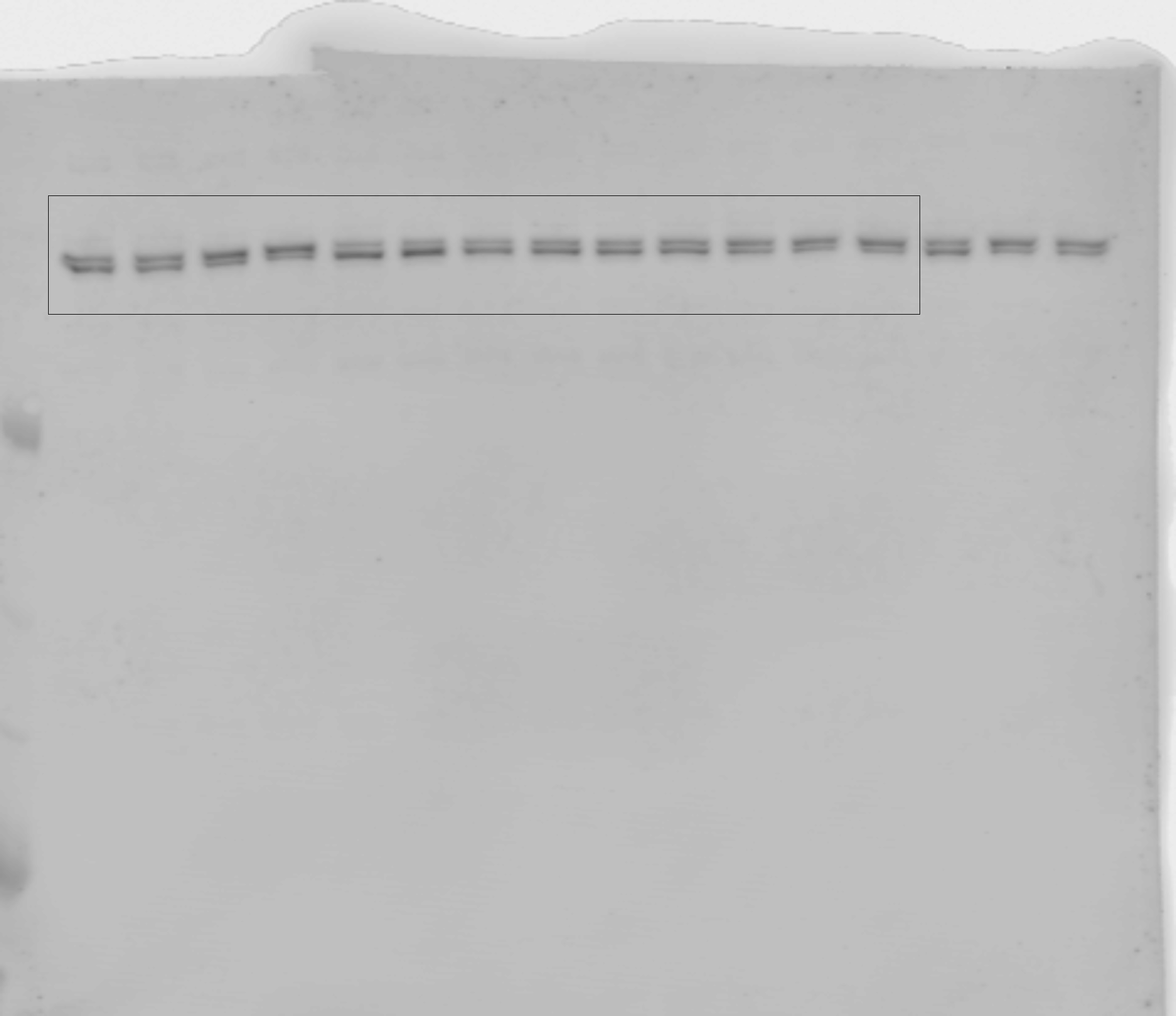

Supplement: Supplementary file 3 — Source data Fig. 1 [file 44318_2025_468_MOESM3_ESM.zip › Figure 1/1G_updated/1G_FANCD2 Western uncropped.tif]

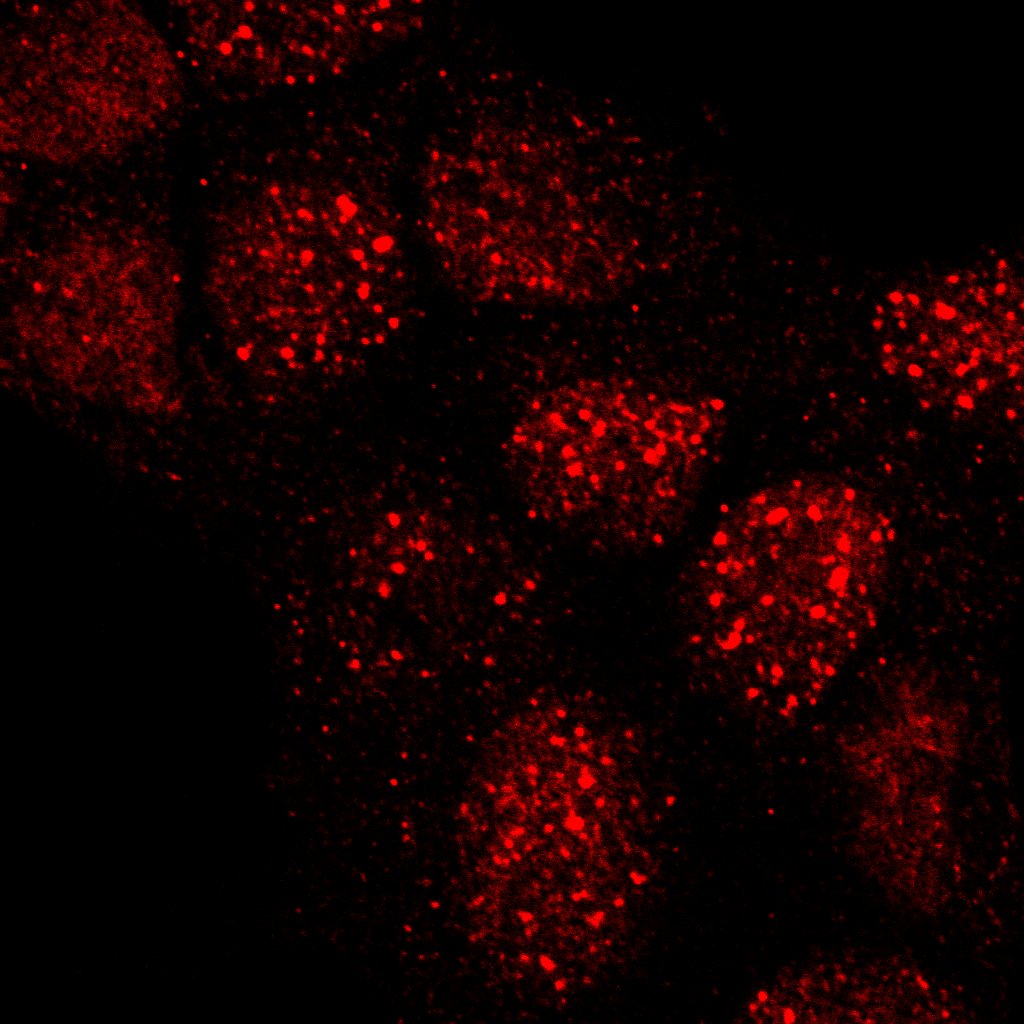

Supplement: Supplementary file 4 — Source data Fig. 4 [file 44318_2025_468_MOESM4_ESM.zip › Figure 4/4C/rSUM_k0521_gH2AX_rFANCD2_WTa.jpg]

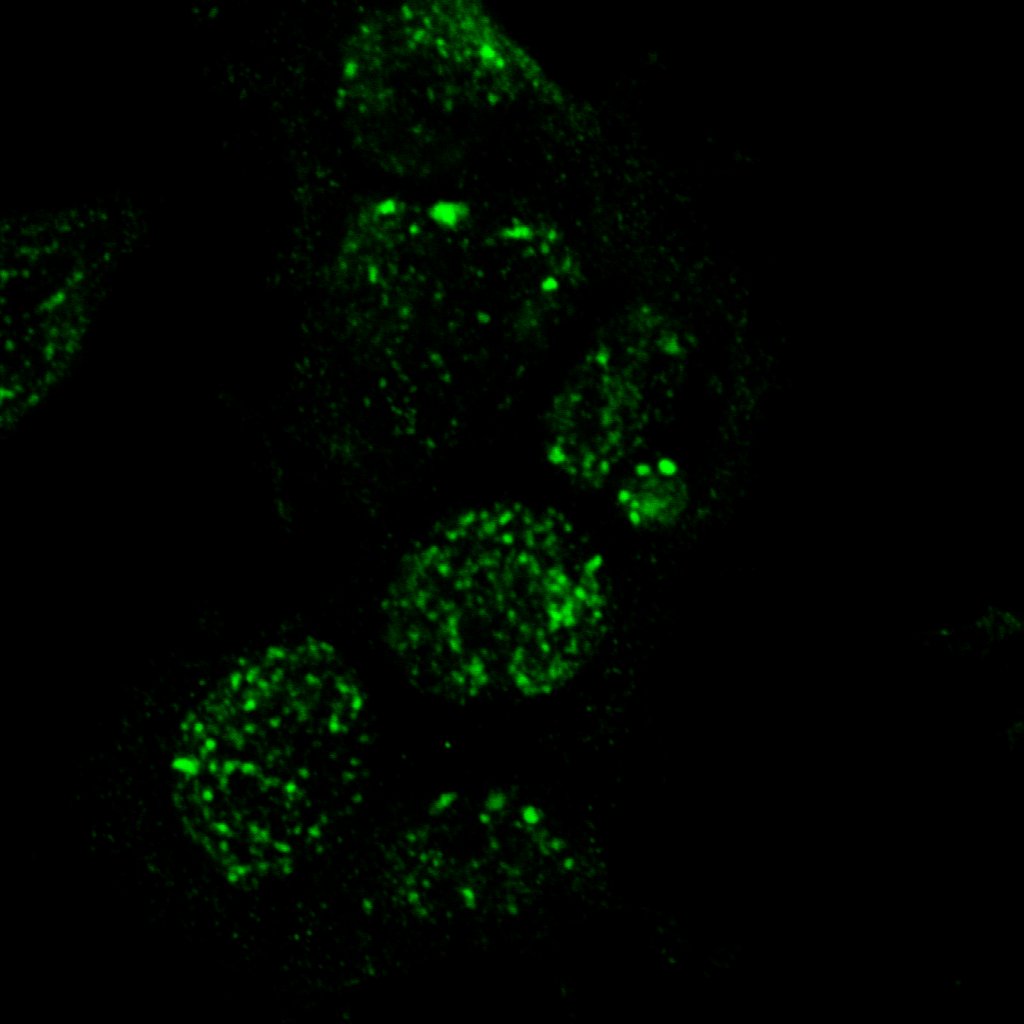

Supplement: Supplementary file 4 — Source data Fig. 4 [file 44318_2025_468_MOESM4_ESM.zip › Figure 4/4C/SUM_k0521_gH2AX_rFANCD2_IDb.jpg]

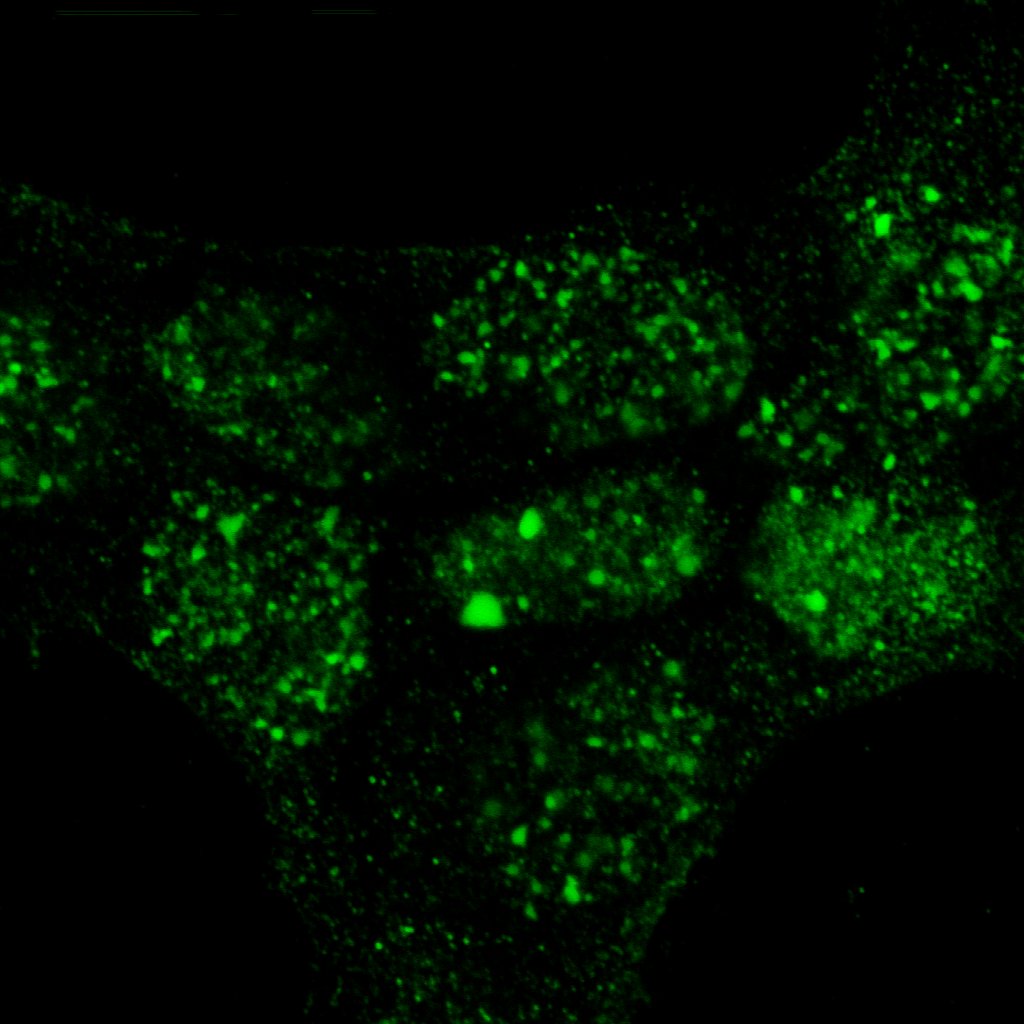

Supplement: Supplementary file 4 — Source data Fig. 4 [file 44318_2025_468_MOESM4_ESM.zip › Figure 4/4C/SUM_k0521_gH2AX_rFANCD2_IDc.jpg]

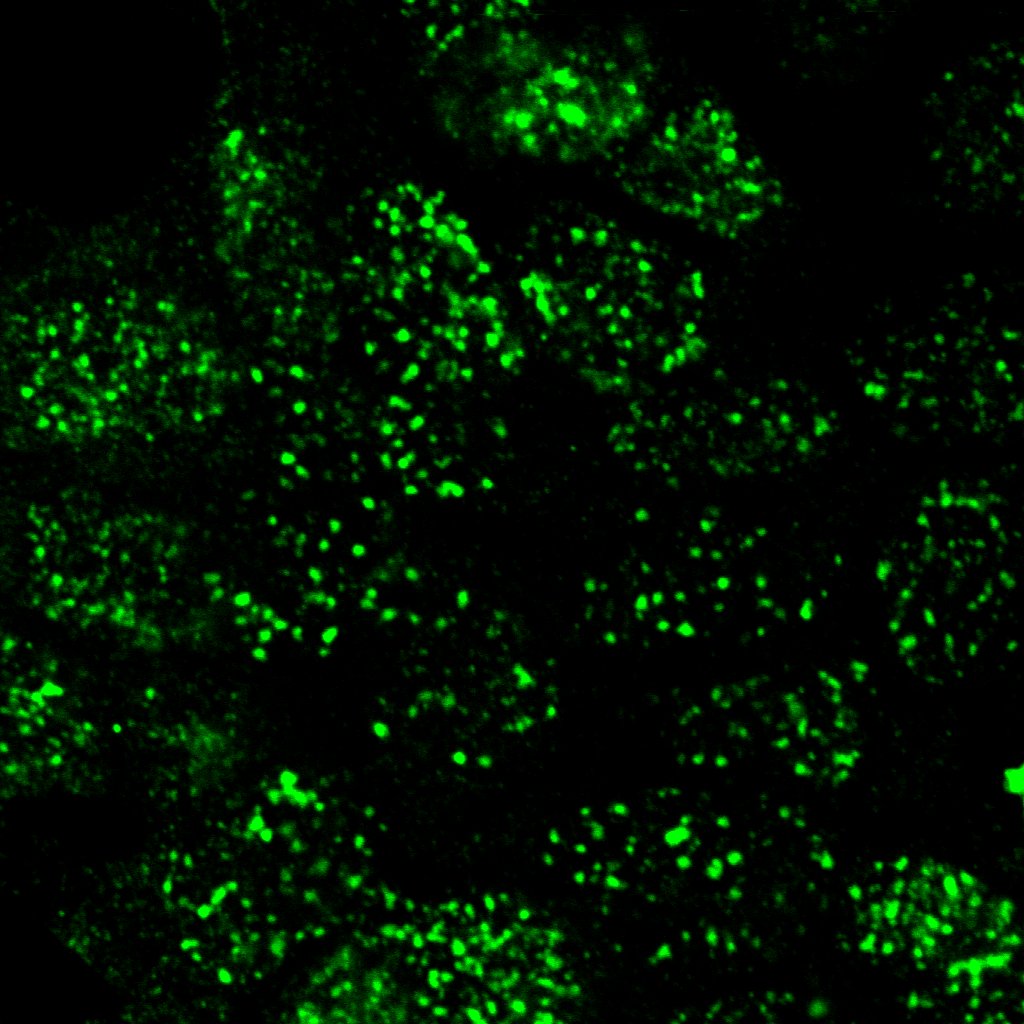

Supplement: Supplementary file 4 — Source data Fig. 4 [file 44318_2025_468_MOESM4_ESM.zip › Figure 4/4C/SUM_k0521_gH2AX_rFANCD2_4Cb.jpg]

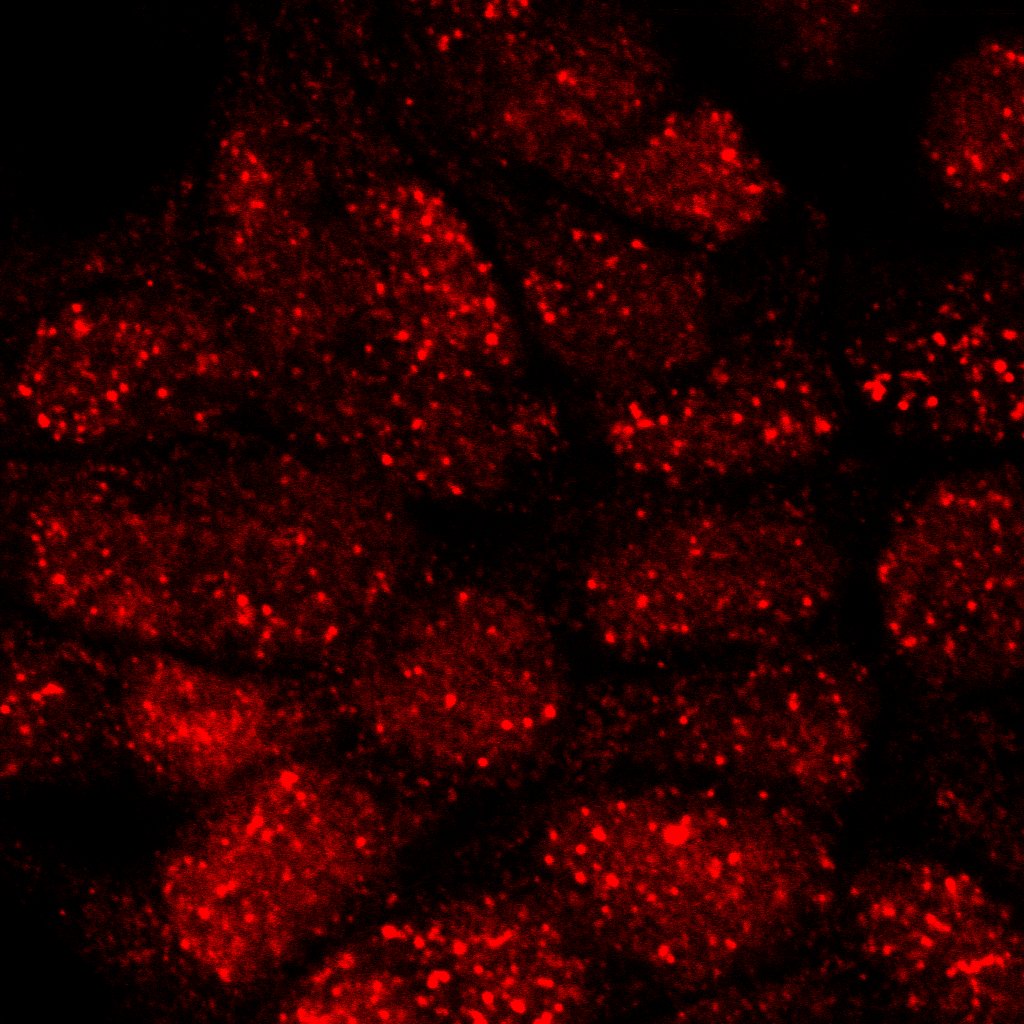

Supplement: Supplementary file 4 — Source data Fig. 4 [file 44318_2025_468_MOESM4_ESM.zip › Figure 4/4C/rSUM_k0521_gH2AX_rFANCD2_4Cb.jpg]

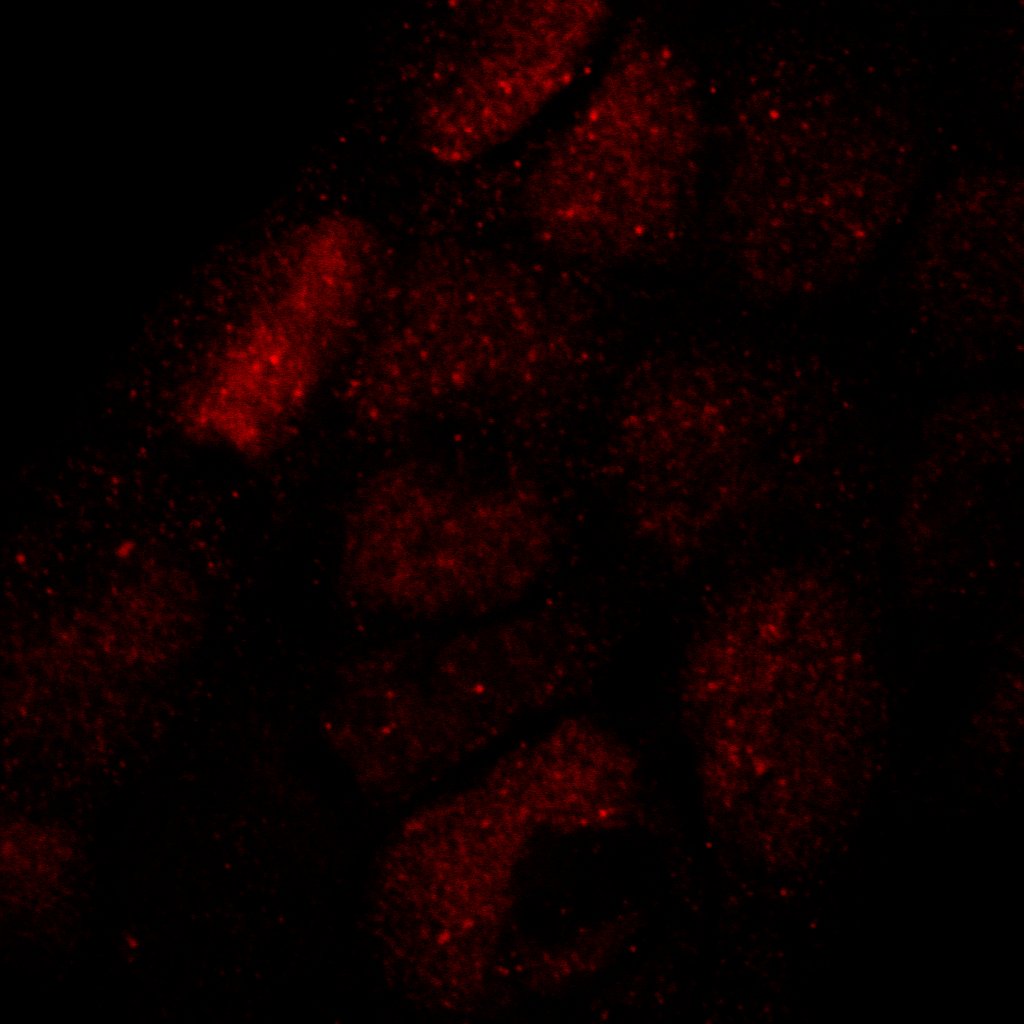

Supplement: Supplementary file 4 — Source data Fig. 4 [file 44318_2025_468_MOESM4_ESM.zip › Figure 4/4C/rSUM_k0521_gH2AX_rFANCD2_8Ma-1.jpg]

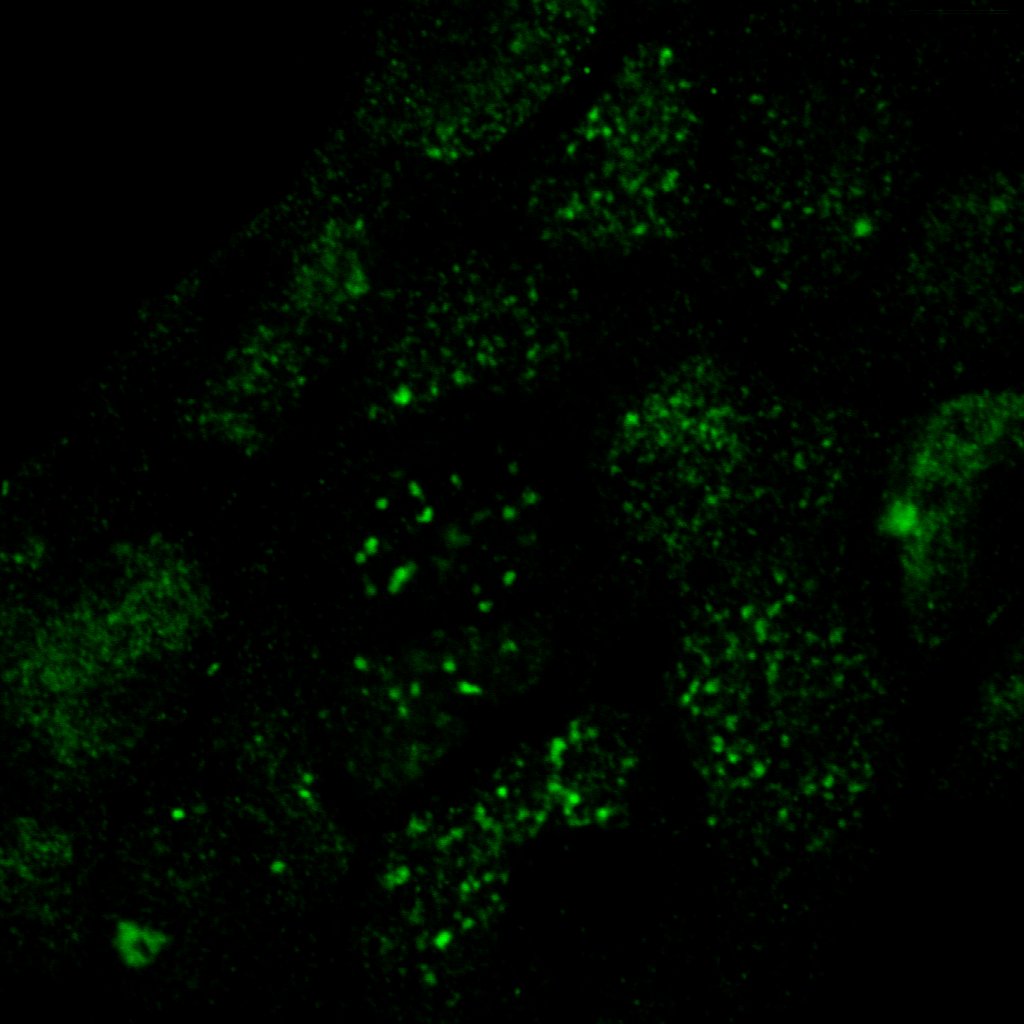

Supplement: Supplementary file 4 — Source data Fig. 4 [file 44318_2025_468_MOESM4_ESM.zip › Figure 4/4C/SUM_k0521_gH2AX_rFANCD2_8Ma-1.jpg]

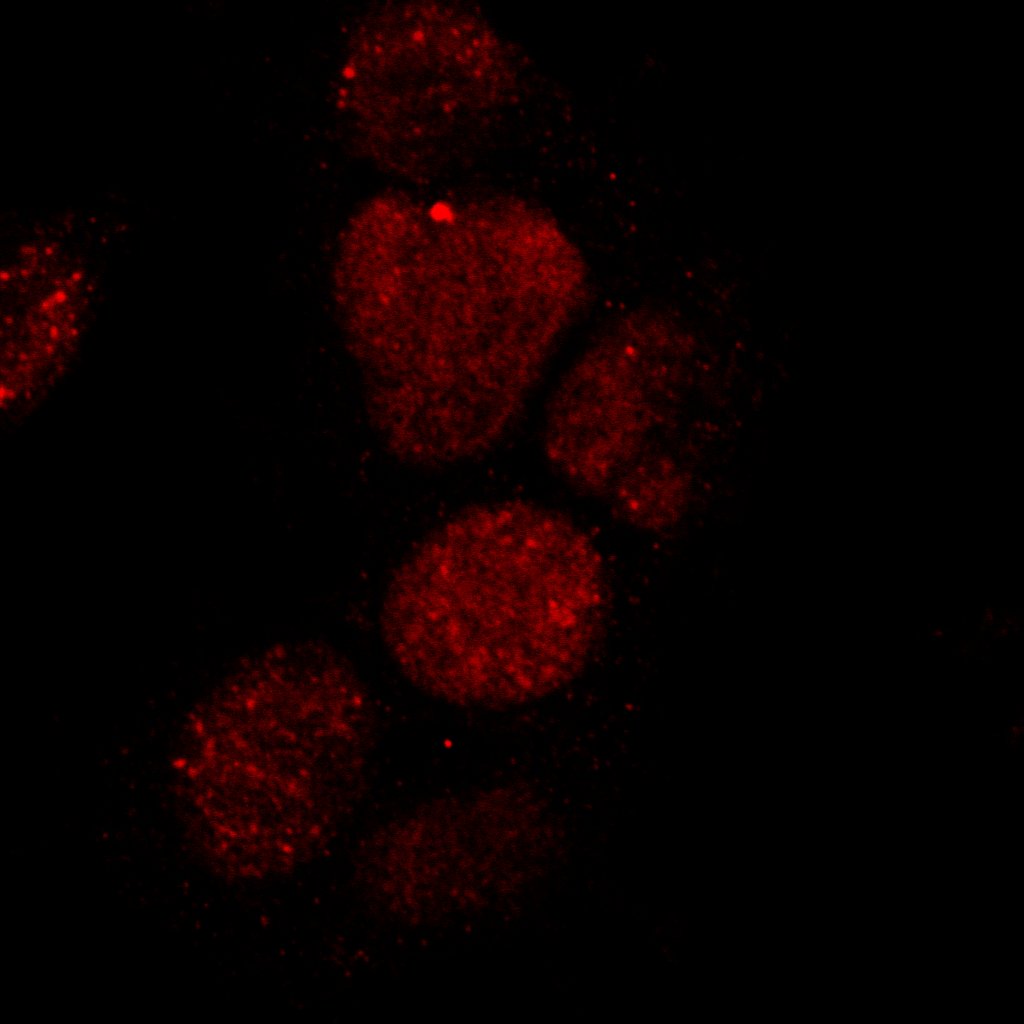

Supplement: Supplementary file 4 — Source data Fig. 4 [file 44318_2025_468_MOESM4_ESM.zip › Figure 4/4C/rSUM_k0521_gH2AX_rFANCD2_IDb.jpg]

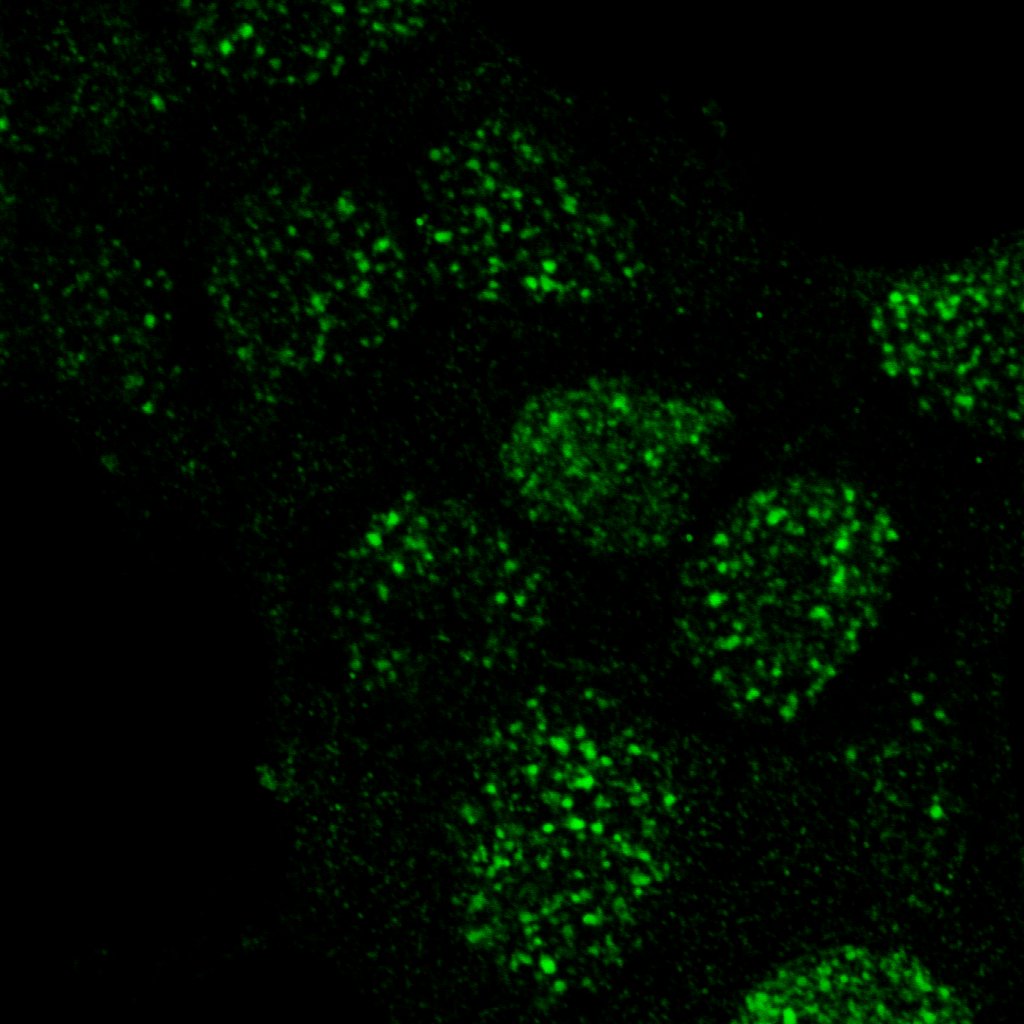

Supplement: Supplementary file 4 — Source data Fig. 4 [file 44318_2025_468_MOESM4_ESM.zip › Figure 4/4C/SUM_k0521_gH2AX_rFANCD2_WTa.jpg]

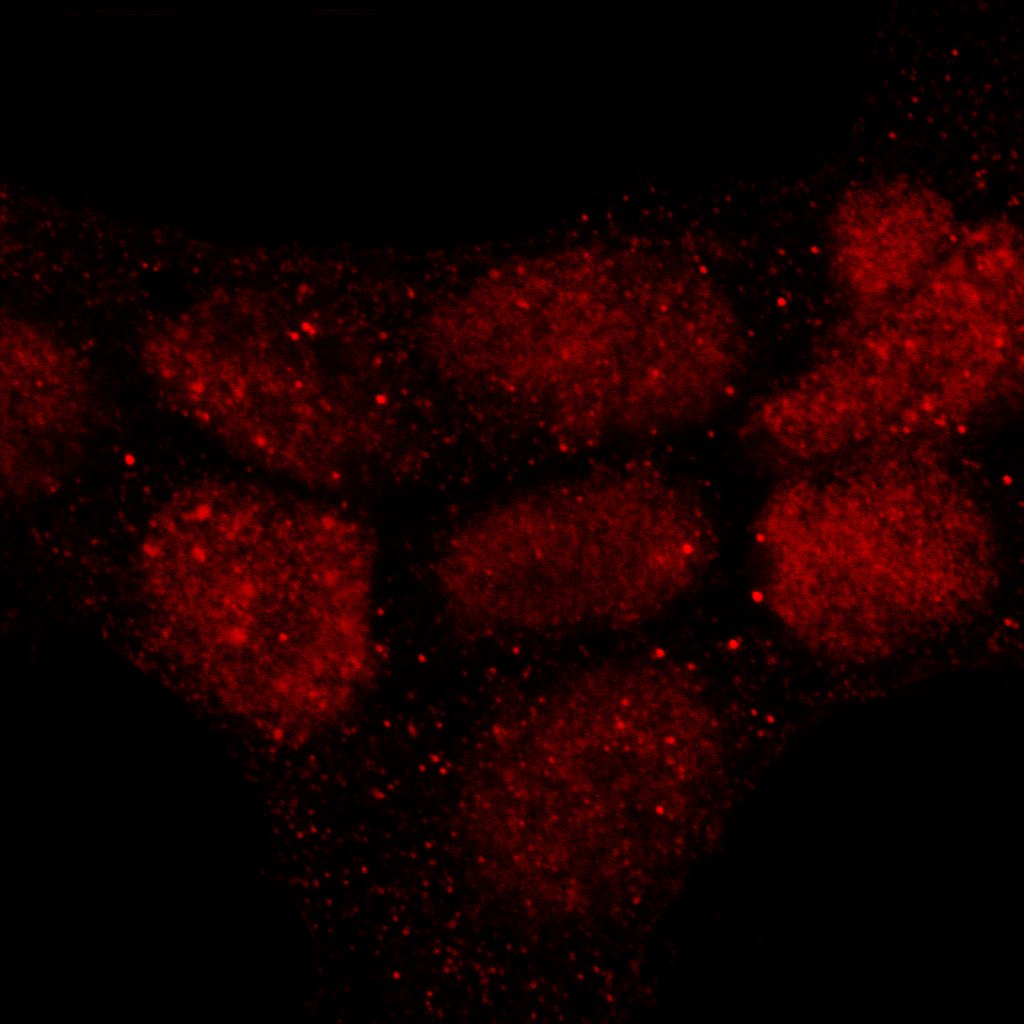

Supplement: Supplementary file 4 — Source data Fig. 4 [file 44318_2025_468_MOESM4_ESM.zip › Figure 4/4C/rSUM_k0521_gH2AX_rFANCD2_IDc.jpg]

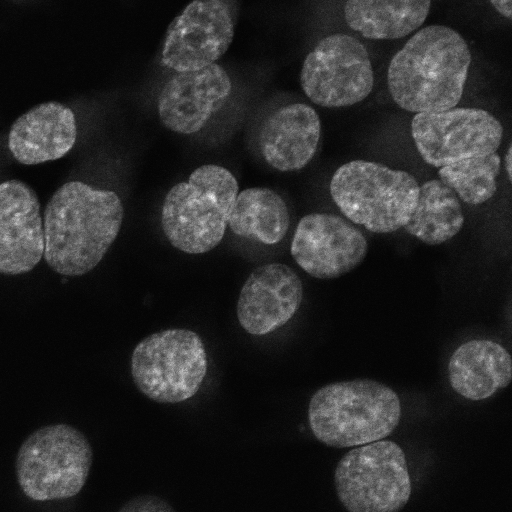

Supplement: Supplementary file 6 — Source data Fig. 6 [file 44318_2025_468_MOESM6_ESM.zip › Figure 6/6F-G/MMC dHel2i_DAPI.tif]

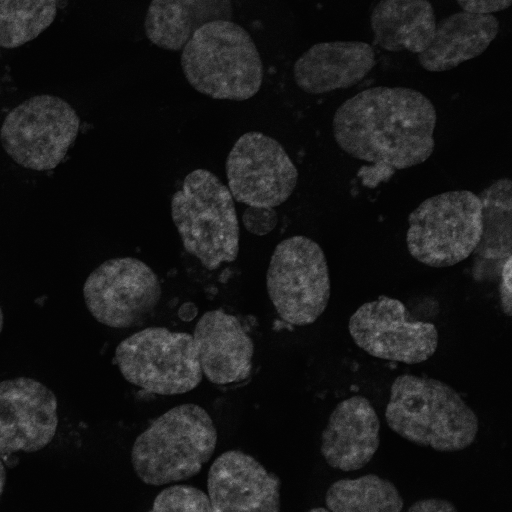

Supplement: Supplementary file 6 — Source data Fig. 6 [file 44318_2025_468_MOESM6_ESM.zip › Figure 6/6F-G/MMC D214A_DAPI.tif]

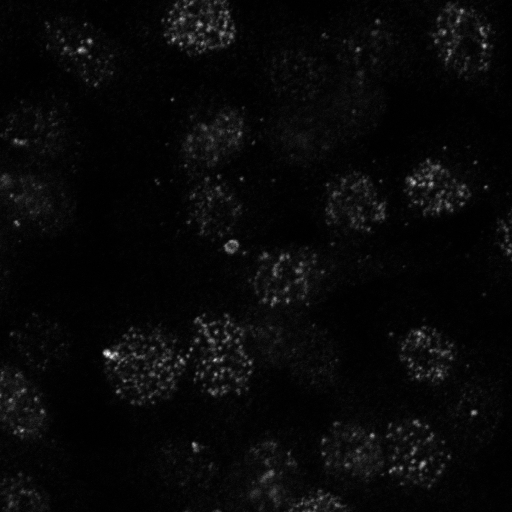

Supplement: Supplementary file 6 — Source data Fig. 6 [file 44318_2025_468_MOESM6_ESM.zip › Figure 6/6F-G/MMC MKO 1_Cy5.tif]

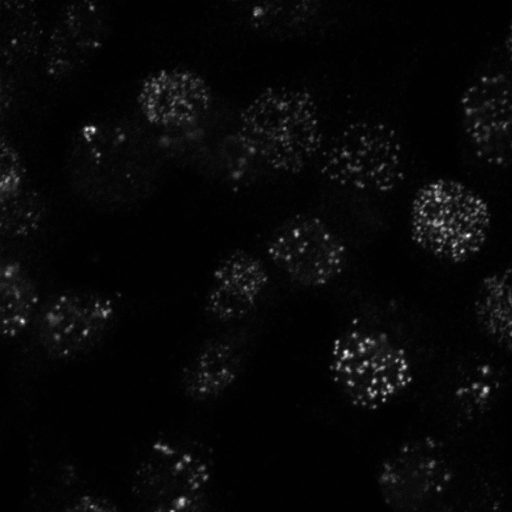

Supplement: Supplementary file 6 — Source data Fig. 6 [file 44318_2025_468_MOESM6_ESM.zip › Figure 6/6F-G/MMC WT 1_Cy5.tif]

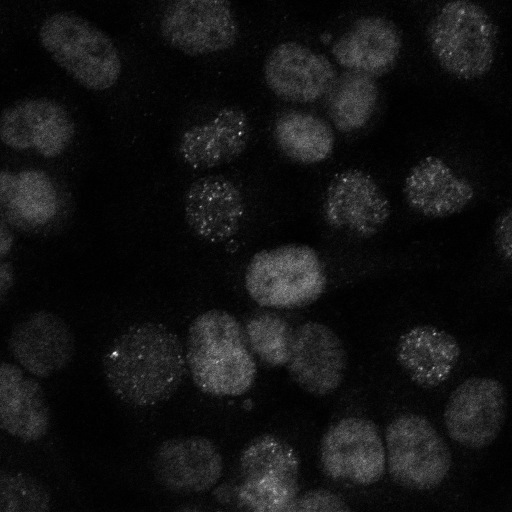

Supplement: Supplementary file 6 — Source data Fig. 6 [file 44318_2025_468_MOESM6_ESM.zip › Figure 6/6F-G/MMC MKO 1_FITC.tif]

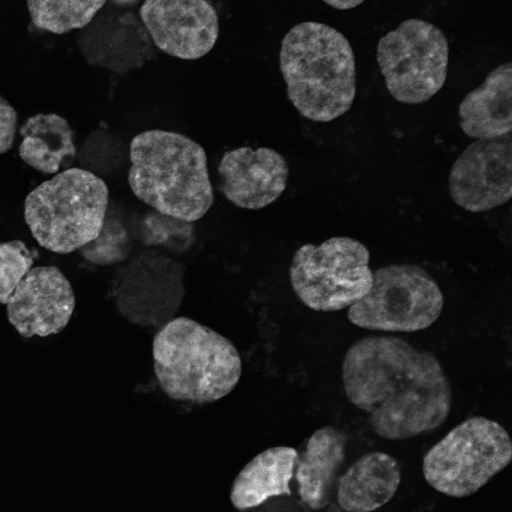

Supplement: Supplementary file 6 — Source data Fig. 6 [file 44318_2025_468_MOESM6_ESM.zip › Figure 6/6F-G/MMC V555F_DAPI.tif]

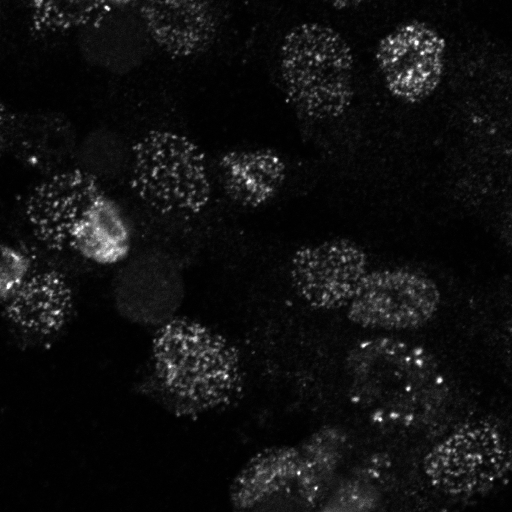

Supplement: Supplementary file 6 — Source data Fig. 6 [file 44318_2025_468_MOESM6_ESM.zip › Figure 6/6F-G/MMC V555F_Cy5.tif]

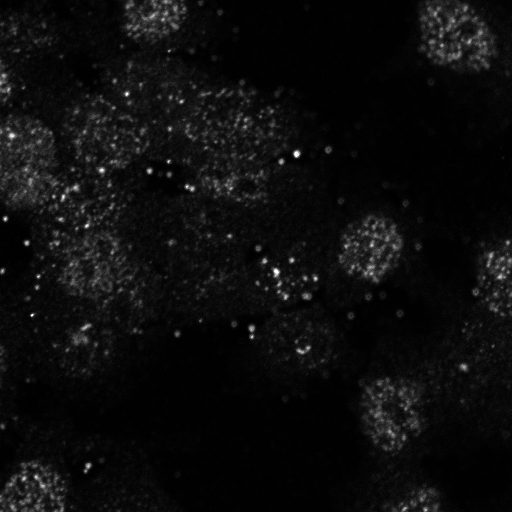

Supplement: Supplementary file 6 — Source data Fig. 6 [file 44318_2025_468_MOESM6_ESM.zip › Figure 6/6F-G/MMC K117R_Cy5.tif]

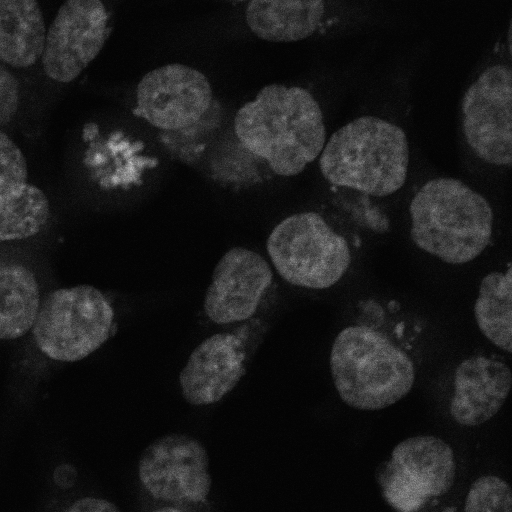

Supplement: Supplementary file 6 — Source data Fig. 6 [file 44318_2025_468_MOESM6_ESM.zip › Figure 6/6F-G/MMC WT 1_DAPI.tif]

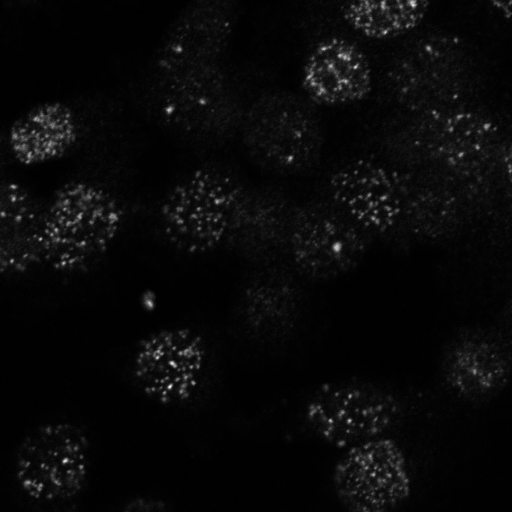

Supplement: Supplementary file 6 — Source data Fig. 6 [file 44318_2025_468_MOESM6_ESM.zip › Figure 6/6F-G/MMC dHel2i_Cy5.tif]

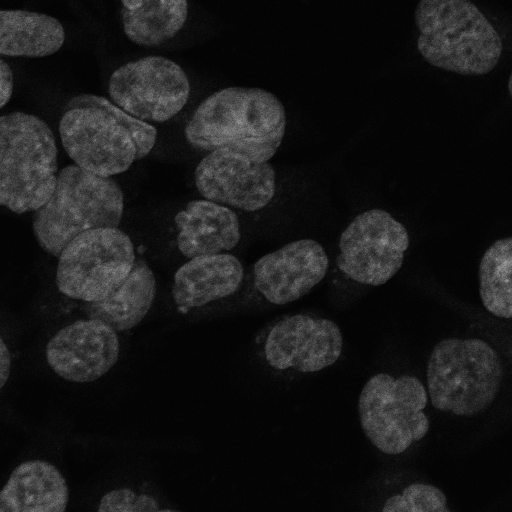

Supplement: Supplementary file 6 — Source data Fig. 6 [file 44318_2025_468_MOESM6_ESM.zip › Figure 6/6F-G/MMC K117R_DAPI.tif]

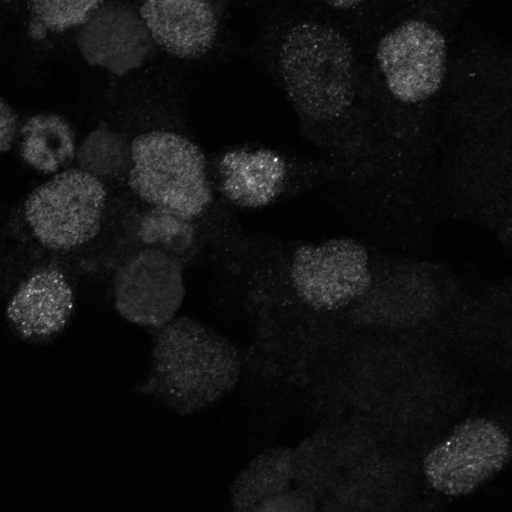

Supplement: Supplementary file 6 — Source data Fig. 6 [file 44318_2025_468_MOESM6_ESM.zip › Figure 6/6F-G/MMC V555F_FITC.tif]

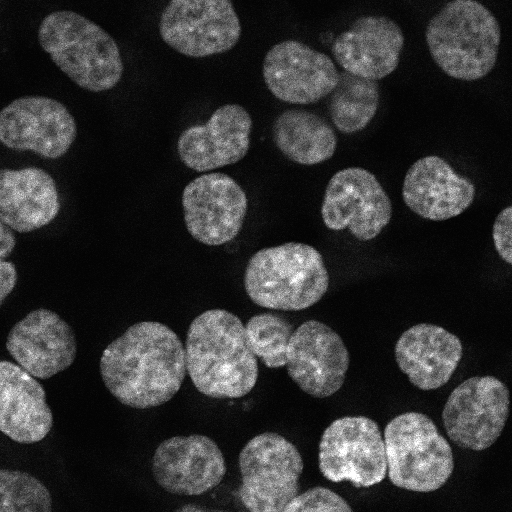

Supplement: Supplementary file 6 — Source data Fig. 6 [file 44318_2025_468_MOESM6_ESM.zip › Figure 6/6F-G/MMC MKO 1_DAPI.tif]

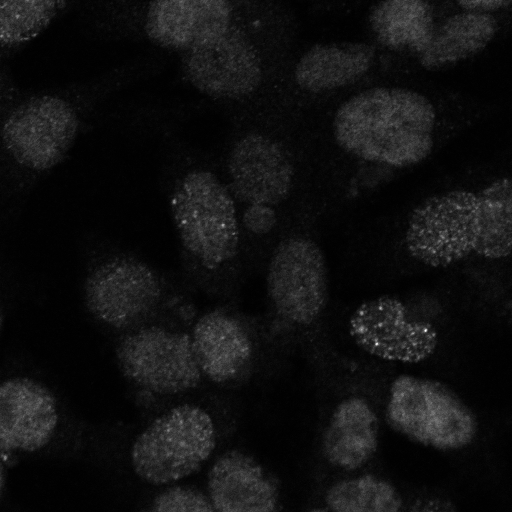

Supplement: Supplementary file 6 — Source data Fig. 6 [file 44318_2025_468_MOESM6_ESM.zip › Figure 6/6F-G/MMC D214A_FITC.tif]

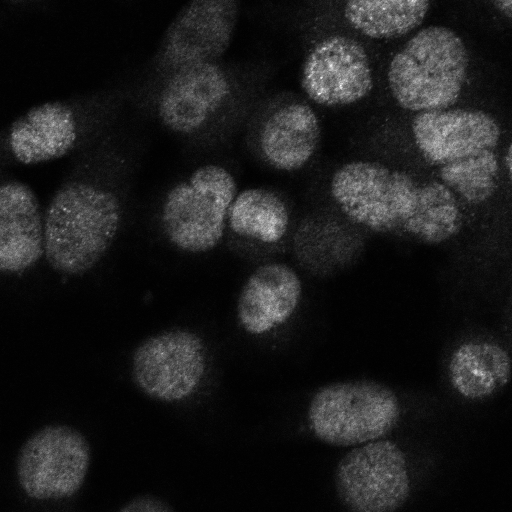

Supplement: Supplementary file 6 — Source data Fig. 6 [file 44318_2025_468_MOESM6_ESM.zip › Figure 6/6F-G/MMC dHel2i_FITC.tif]

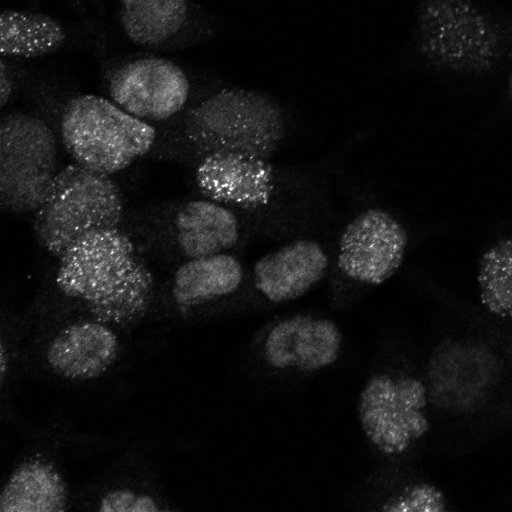

Supplement: Supplementary file 6 — Source data Fig. 6 [file 44318_2025_468_MOESM6_ESM.zip › Figure 6/6F-G/MMC K117R_FITC.tif]

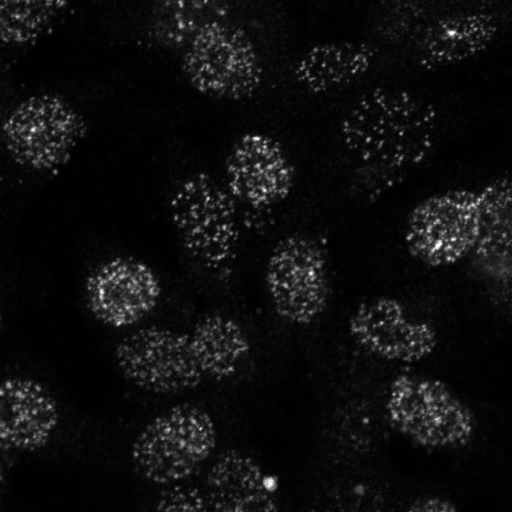

Supplement: Supplementary file 6 — Source data Fig. 6 [file 44318_2025_468_MOESM6_ESM.zip › Figure 6/6F-G/MMC D214A_Cy5.tif]

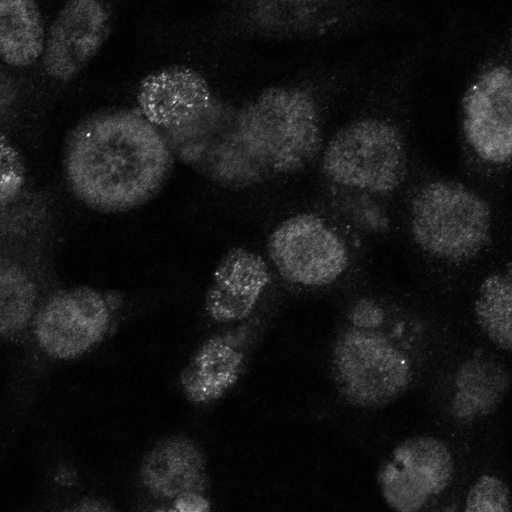

Supplement: Supplementary file 6 — Source data Fig. 6 [file 44318_2025_468_MOESM6_ESM.zip › Figure 6/6F-G/MMC WT 1_FITC.tif]

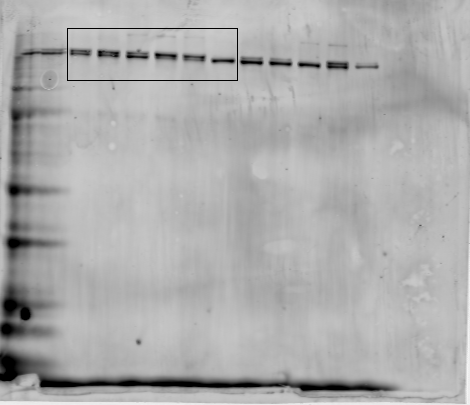

Supplement: Supplementary file 7 — Source data Fig. 7 [file 44318_2025_468_MOESM7_ESM.zip › Figure 7/7C_updated/7C_FANCD2 uncropped.tif]

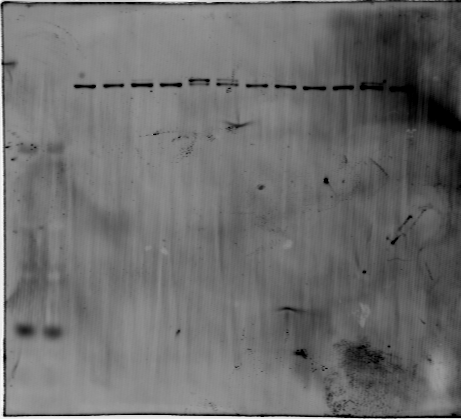

Supplement: Supplementary file 7 — Source data Fig. 7 [file 44318_2025_468_MOESM7_ESM.zip › Figure 7/7C_updated/7C_FANCI uncropped.tif]

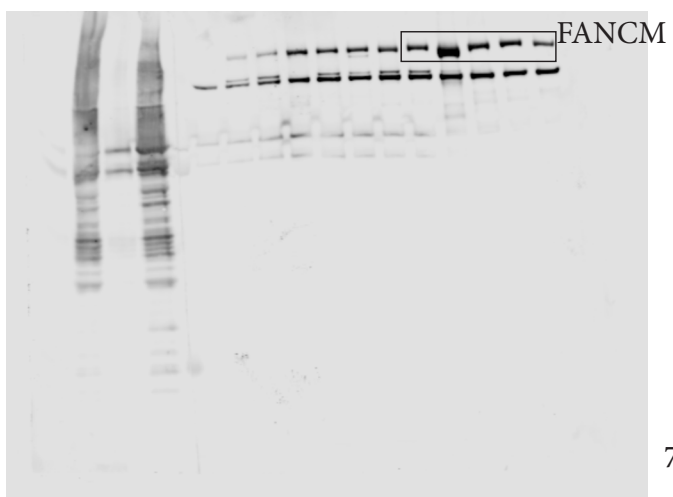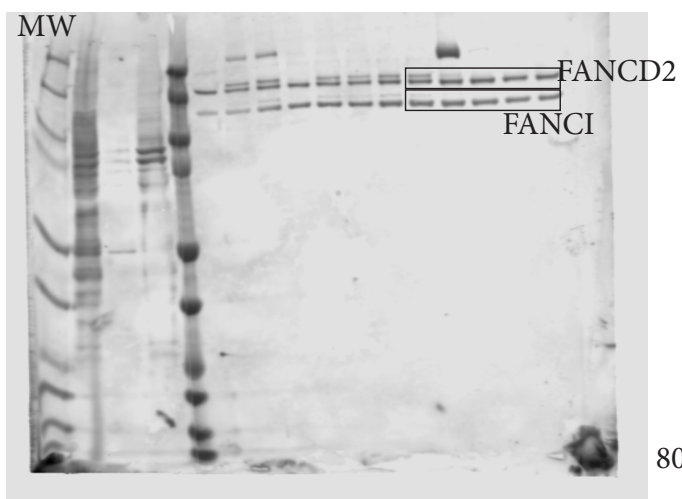

Supplement: Supplementary file 7 — Source data Fig. 7 [file 44318_2025_468_MOESM7_ESM.zip › Figure 7/7F/FANCM uncropped 7F.pdf]

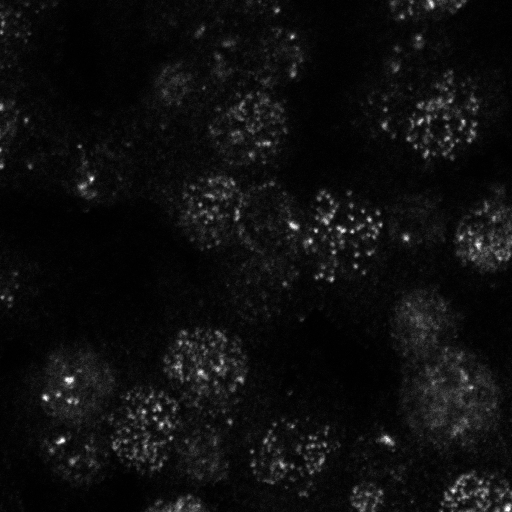

Supplement: Supplementary file 7 — Source data Fig. 7 [file 44318_2025_468_MOESM7_ESM.zip › Figure 7/7G-H/MMC E964K_Cy5.tif]

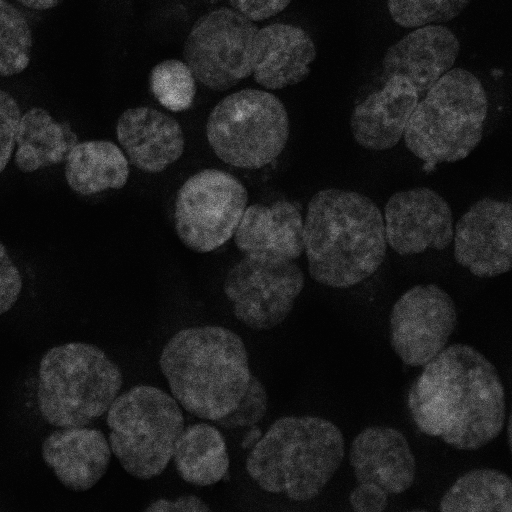

Supplement: Supplementary file 7 — Source data Fig. 7 [file 44318_2025_468_MOESM7_ESM.zip › Figure 7/7G-H/MMC E964K_DAPI.tif]

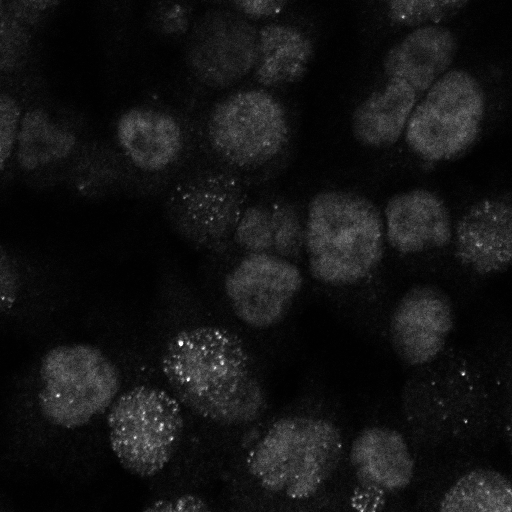

Supplement: Supplementary file 7 — Source data Fig. 7 [file 44318_2025_468_MOESM7_ESM.zip › Figure 7/7G-H/MMC E964K_FITC.tif]

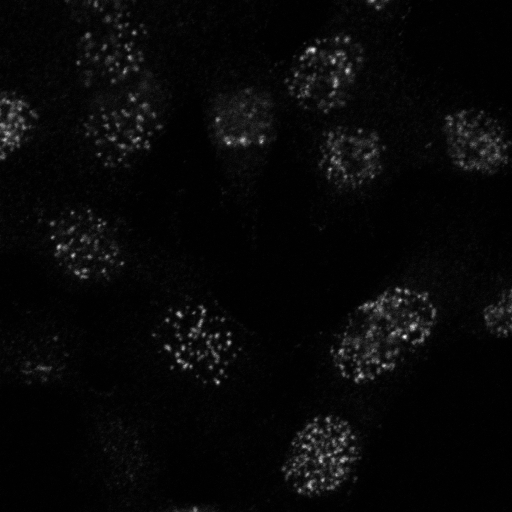

Supplement: Supplementary file 7 — Source data Fig. 7 [file 44318_2025_468_MOESM7_ESM.zip › Figure 7/7G-H/MMC MKO 2_Cy5.tif]

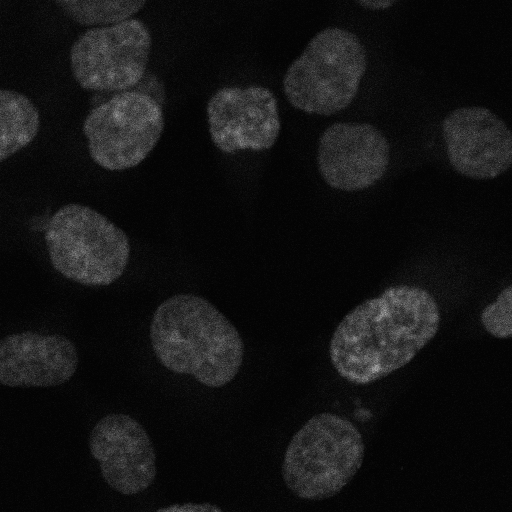

Supplement: Supplementary file 7 — Source data Fig. 7 [file 44318_2025_468_MOESM7_ESM.zip › Figure 7/7G-H/MMC MKO 2_DAPI.tif]

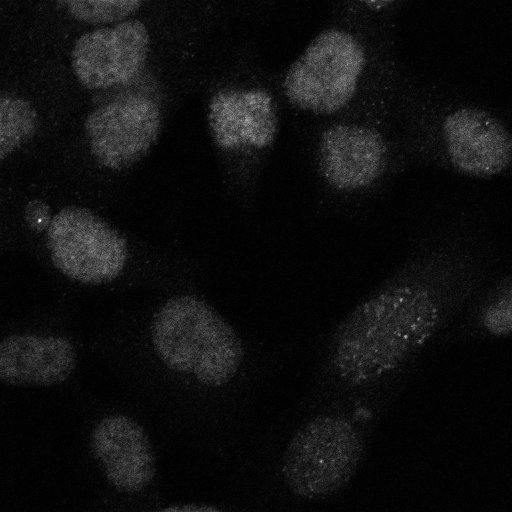

Supplement: Supplementary file 7 — Source data Fig. 7 [file 44318_2025_468_MOESM7_ESM.zip › Figure 7/7G-H/MMC MKO 2_FITC.tif]

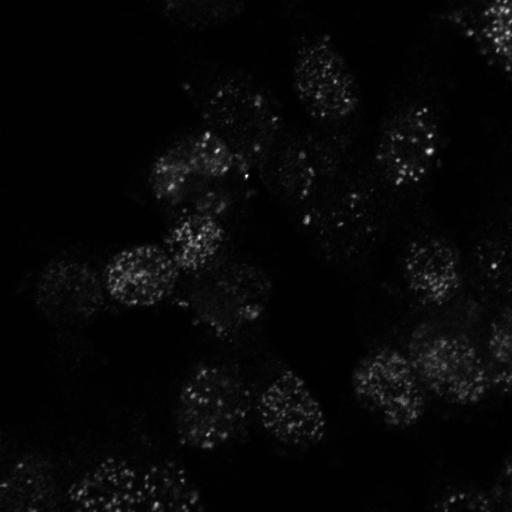

Supplement: Supplementary file 7 — Source data Fig. 7 [file 44318_2025_468_MOESM7_ESM.zip › Figure 7/7G-H/MMC MM1_Cy5.tif]

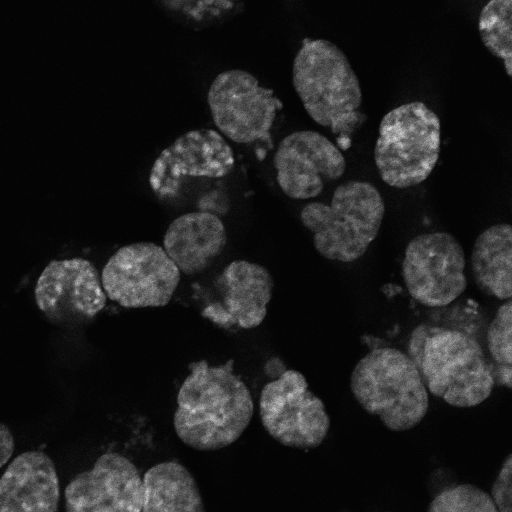

Supplement: Supplementary file 7 — Source data Fig. 7 [file 44318_2025_468_MOESM7_ESM.zip › Figure 7/7G-H/MMC MM1_DAPI.tif]

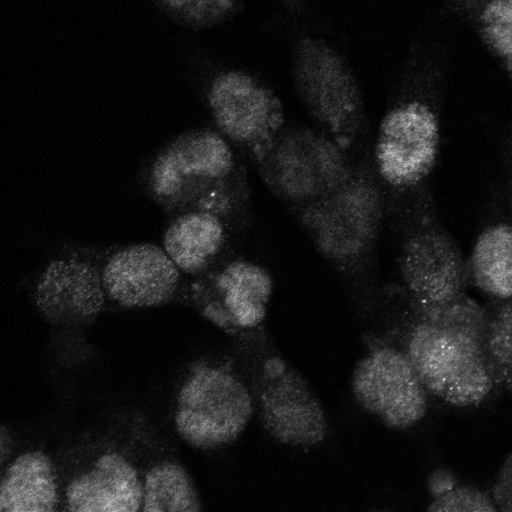

Supplement: Supplementary file 7 — Source data Fig. 7 [file 44318_2025_468_MOESM7_ESM.zip › Figure 7/7G-H/MMC MM1_FITC.tif]

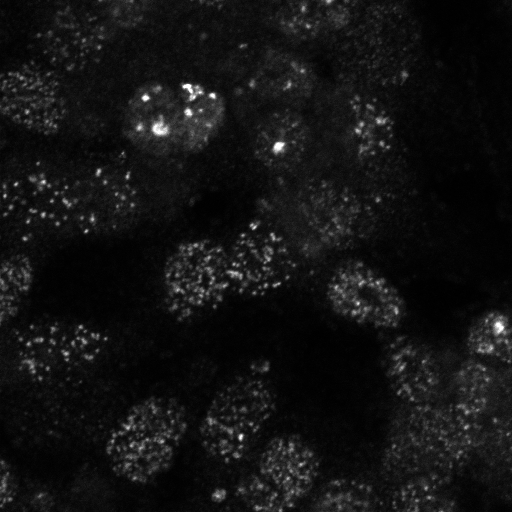

Supplement: Supplementary file 7 — Source data Fig. 7 [file 44318_2025_468_MOESM7_ESM.zip › Figure 7/7G-H/MMC P999A_Cy5.tif]

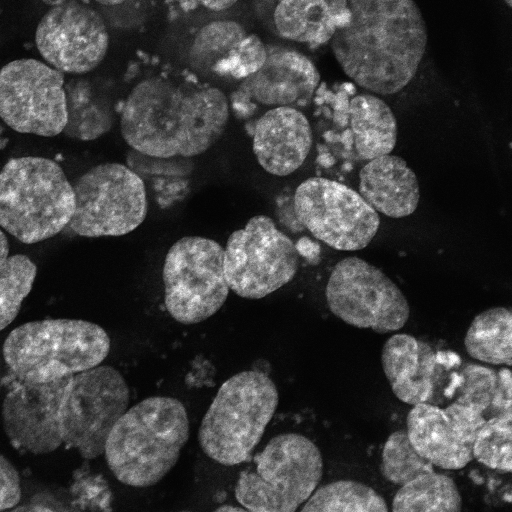

Supplement: Supplementary file 7 — Source data Fig. 7 [file 44318_2025_468_MOESM7_ESM.zip › Figure 7/7G-H/MMC P999A_DAPI.tif]

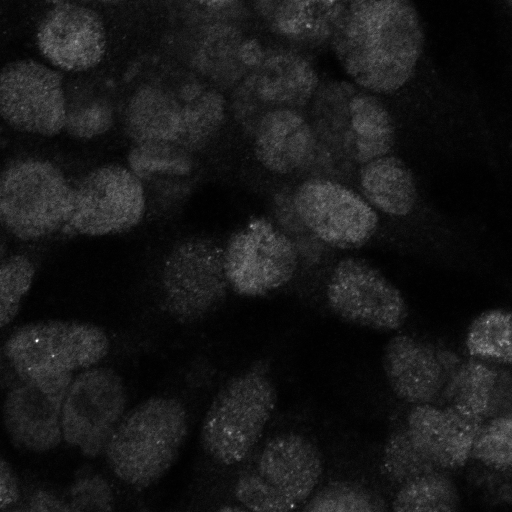

Supplement: Supplementary file 7 — Source data Fig. 7 [file 44318_2025_468_MOESM7_ESM.zip › Figure 7/7G-H/MMC P999A_FITC.tif]

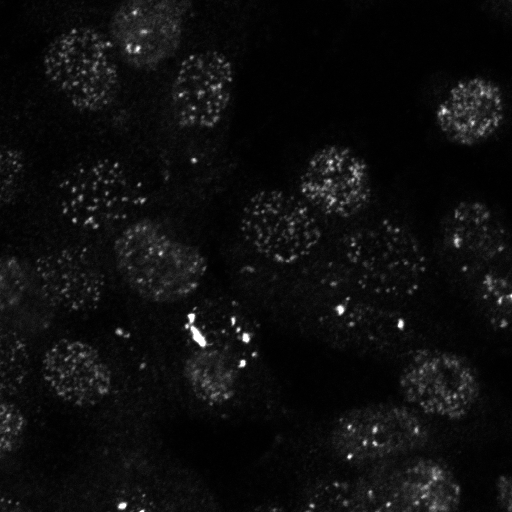

Supplement: Supplementary file 7 — Source data Fig. 7 [file 44318_2025_468_MOESM7_ESM.zip › Figure 7/7G-H/MMC V990A_Cy5.tif]

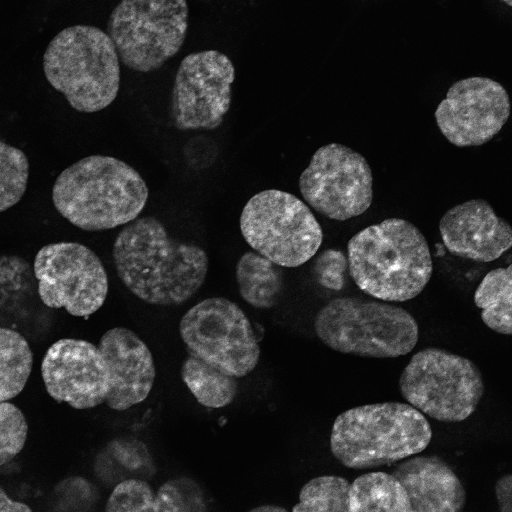

Supplement: Supplementary file 7 — Source data Fig. 7 [file 44318_2025_468_MOESM7_ESM.zip › Figure 7/7G-H/MMC V990A_DAPI.tif]

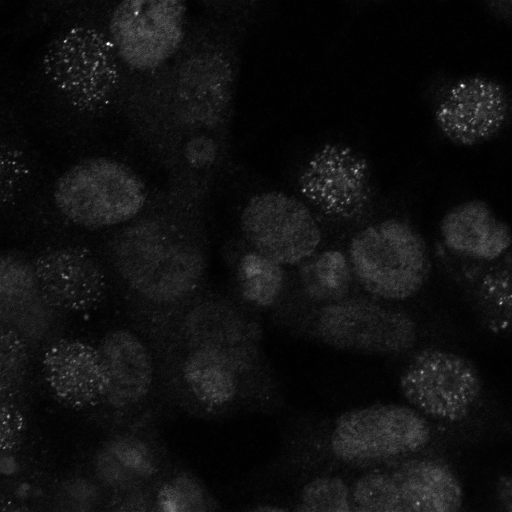

Supplement: Supplementary file 7 — Source data Fig. 7 [file 44318_2025_468_MOESM7_ESM.zip › Figure 7/7G-H/MMC V990A_FITC.tif]

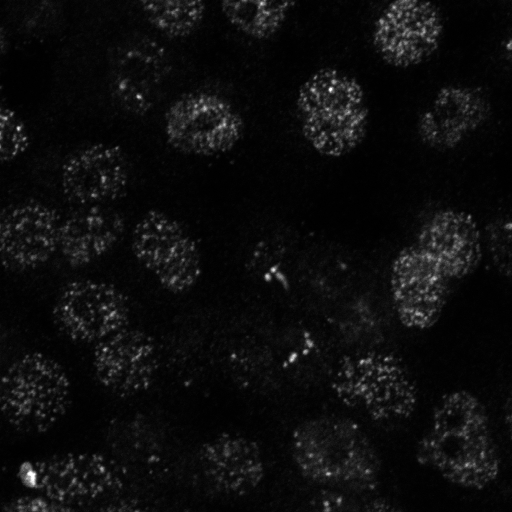

Supplement: Supplementary file 7 — Source data Fig. 7 [file 44318_2025_468_MOESM7_ESM.zip › Figure 7/7G-H/MMC WT 2_Cy5.tif]

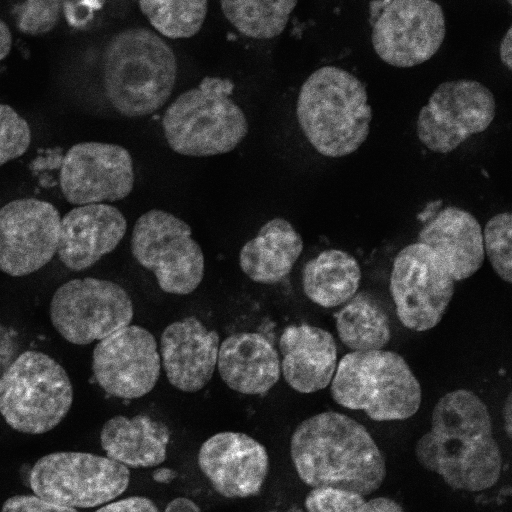

Supplement: Supplementary file 7 — Source data Fig. 7 [file 44318_2025_468_MOESM7_ESM.zip › Figure 7/7G-H/MMC WT 2_DAPI.tif]

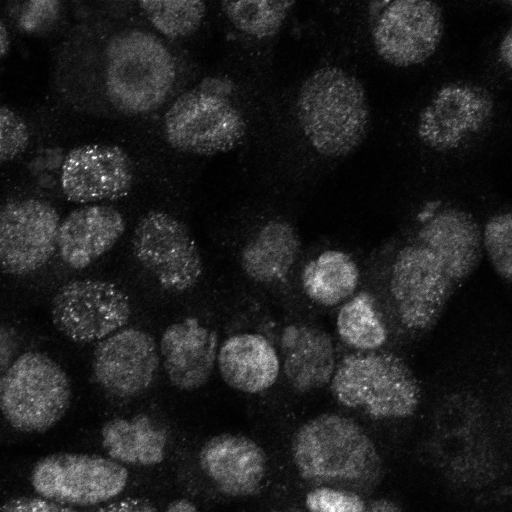

Supplement: Supplementary file 7 — Source data Fig. 7 [file 44318_2025_468_MOESM7_ESM.zip › Figure 7/7G-H/MMC WT 2_FITC.tif]
