# Supplementary material for: Structural basis of Fanconi anemia pathway activation by FANCM
Source: EMBO J. 2025 May 30;44(14):4013–36. doi: 10.1038/s44318-025-00468-3 (PMC12263834; doi:10.1038/s44318-025-00468-3)
Supplement: Supplementary file 8 — Expanded View Figures [file 44318_2025_468_MOESM8_ESM.pdf]

Expanded View Figures

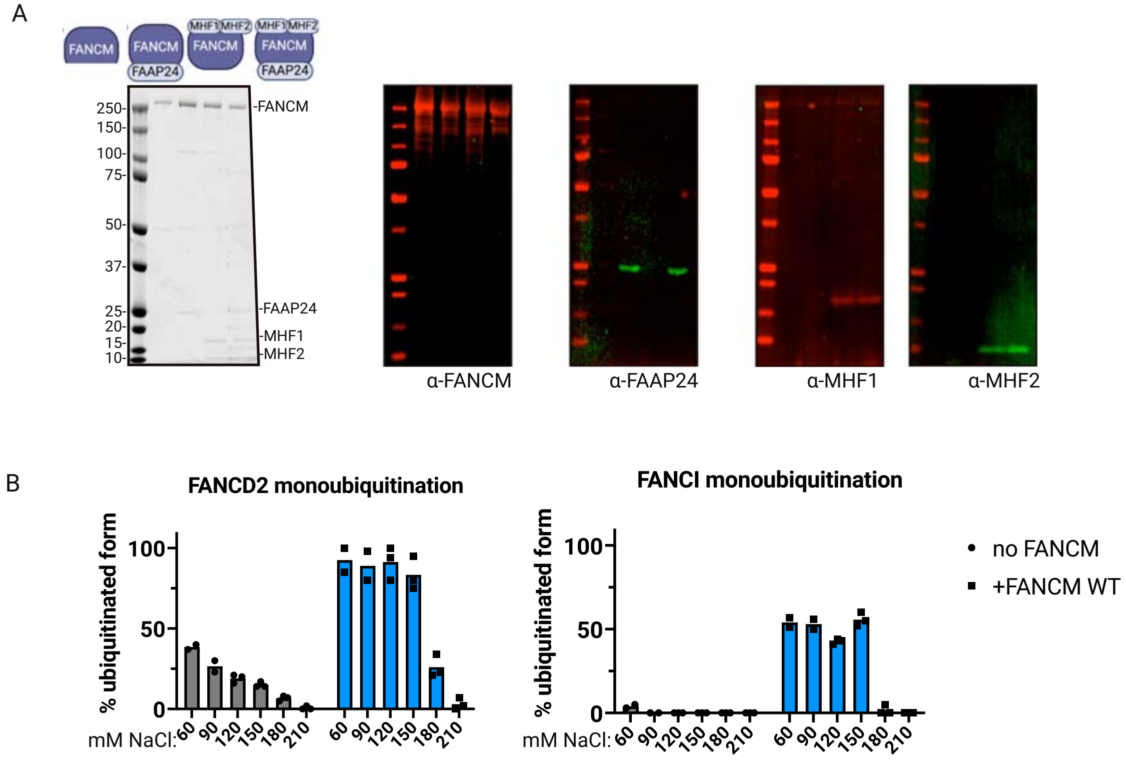

Figure EV1. Purified FANCM complexes overcome the salt inhibition of FANCD2 and FANCI monoubiquitination by FA core complex.

(**A**) Purified FANCM complexes, shown by Coomassie blue staining (as per Fig. 1B). Western blotting reveals that each subunit is present. (**B**) FANCD2:FANCI monoubiquitination reactions conducted in increasing concentrations (60, 90, 120, 150, 180 and 210 nM) NaCl without FANCM (gray bars) or with 100 nM FANCM (blue bars). Reactions were stopped at 30 min and ubiquitinated form was quantified. Results from 2 (60 and 90 nM) or 3 (other concentrations) independent experiments, individual values, mean ± SD shown.

 © The Author(s)

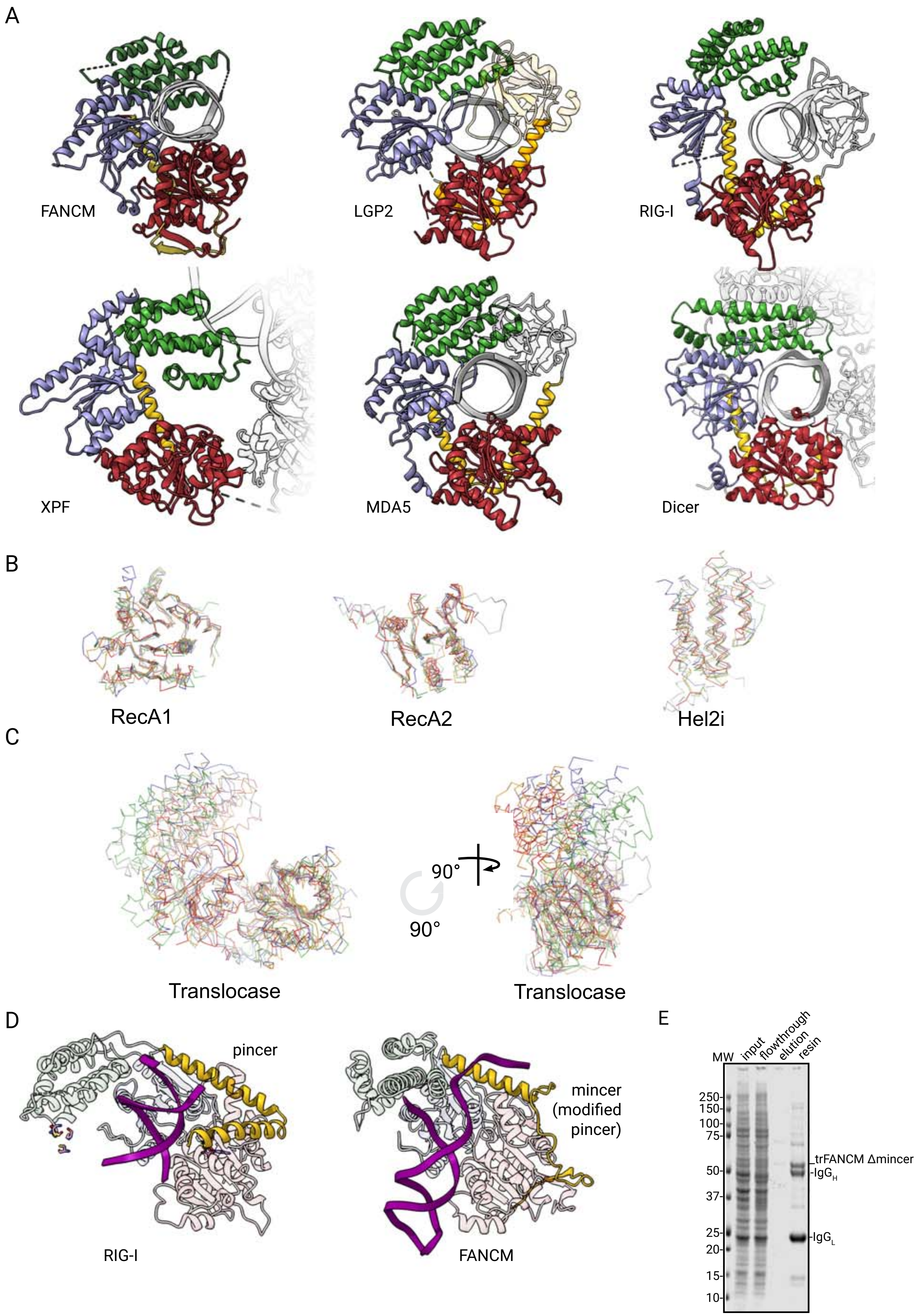
A
FANCM
LGP2
RIG-I
XPF
MDA5
Dicer
B
RecA1
RecA2
Hel2i
C
90°
90°
Translocase
Translocase
D
pincer
mincer
(modified
pincer)
RIG-I
FANCM
E
MW
input
flowthrough
elution
resin
250-
150-
100-
75-
50-
37-
25-
20-
15-
10-
trFANCM Δmincer
IgG$_H$
IgG$_L$

Figure EV2. Similarities and differences between FANCM translocase domain and structurally related proteins.

(**A**) Related translocase domains with structures in the PDB: LGP2 1–482 (Uchikawa et al, 2016), RIG-I 236–744 (Luo et al, 2011), XPF 1–631 (Jones et al, 2020), MDA-5 308–836 (Yu et al, 2018) and Dicer 8-508 (Deng et al, 2023). All related structures are bound to dsRNA and no ATP analog (except for XPF which is bound to dsDNA). (**B**) Domains were superposed using "SUPERPOSE" from within the CCP4 program suite, using secondary structure matching for the specified residue ranges. Structures are depicted in ribbon representation in pymol software. FANCM = red, LGP2 = orange, RIG-I = green, MDA-5 = blue, Dicer = gray. (**C**) full translocase domains overlayed (XPF excluded) superposed using the RecA2 domain, coloring as in (**B**, **D**) specific orientation of RIG-I and FANCM highlighting the "mincer" domain of FANCM compared to the "pincer" domain of RIG-I. (**E**) trFANCM-Δmincer (FANCM 80–590) is mostly insoluble and remains stuck on resin during Flag affinity purification.

 © The Author(s)

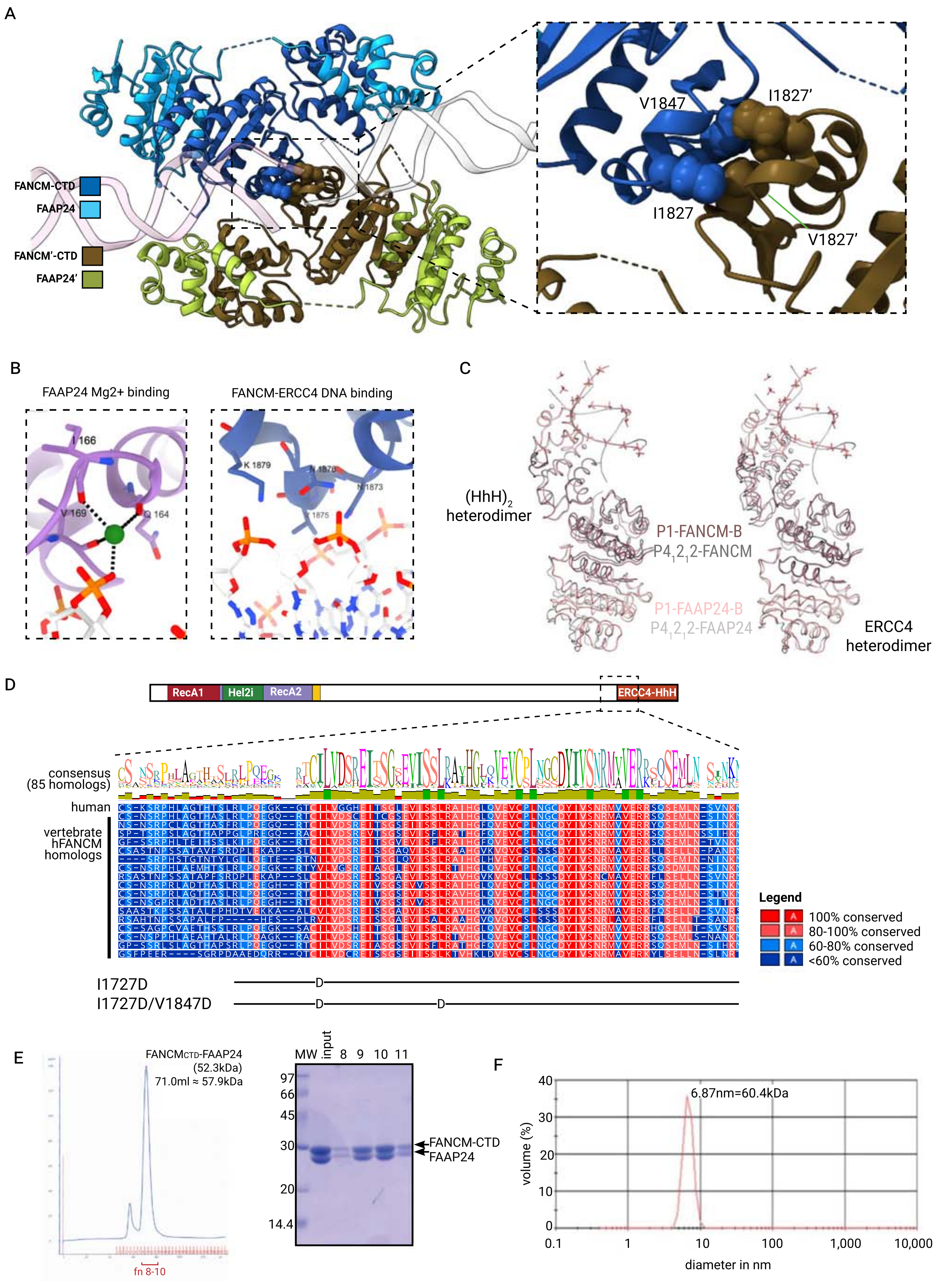
A
FANCM-CTD
FAAP24
FANCM′-CTD
FAAP24′
V1847
I1827′
I1827
V1827′
B
FAAP24 Mg2+ binding
FANCM-ERCC4 DNA binding
C
(HhH)2 heterodimer
P1-FANCM-B
P41212-FANCM
P1-FAAP24-B
P41212-FAAP24
ERCC4 heterodimer
D
RecA1
Hel2i
RecA2
ERCC4-HhH
consensus (85 homologs)
human
vertebrate hFANCM homologs
Legend
100% conserved
80-100% conserved
60-80% conserved
<60% conserved
I1727D
I1727D/V1847D
E
FANCMCTD-FAAP24 (52.3kDa)
71.0ml ≈ 57.9kDa
fn 8-10
MW input 8 9 10 11
97
66
45
30
20
14.4
FANCM-CTD
FAAP24
F
6.87nm=60.4kDa
volume (%)
diameter in nm
0.1
1
10
100
1,000
10,000

© The Author(s)

Figure EV3. Detailed view of FANCM-CTD:FAAP24 structure and conservation.

(**A**) Dimeric arrangement of FANCM-CTD:FAAP24 complexes in P4,2,2 crystal. The interface involves helix 1 and 2 and beta-strand 2 of the two different molecules, which form an alternative unit cell, and residues at the interface are highly conserved within FANCM. Key residues include I1827, which is highly conserved in FANCM, packing against V1847 and P1849. The interface has 2-fold non-crystallographic symmetry, and in general residues at this interface have a highly similar positions and the same rotamer on each side of the interface. The one exception is R1838, which is only modeled in copy B. (**B**) zoom in of left:FAAP24 (Mauve) and Mg2+ (green) binding site, and right: additional FANCM-DNA-binding site. Key residues shown by numbering. Phosphate backbone of DNA in orange/red. Refer to main text for details. (**C**) Superposition using the (HhH)2 and ERCC4 heterodimers separately shows that the orientation of the two domains is slightly different in the two crystal forms, suggesting that the ERCC4-$(HhH)_2$ interface may be subtly different. This change in orientation is, however, very slight and the DynDom server (Hayward and Berendsen 1998), which allows easy calculation of domain movements in different crystal forms was unable to detect it. The core hydrophobic residues involved in this intramolecular FANCM interface are identical, consistent with it being a conserved feature present in solution. (**D**) Conservation of the region of FANCM ERCC4 domain N-terminal boundary across 85 vertebrate homologs using the consurf server (Ashkenazy et al, 2016). (**E**) Size exclusion chromatography reveals a peak corresponding to size of heterodimer. (**F**) Dynamic light scattering (DLS) shows size expected for a heterodimer in solution.

 © The Author(s)

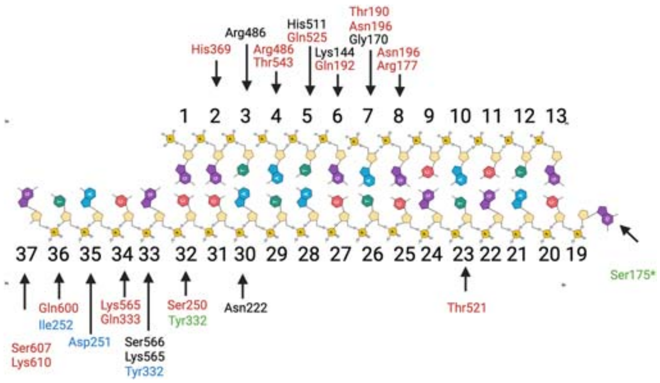

Figure EV4. FANCM translocase contacts with DNA.

DNA bases are numbered from 5′ to 3′ end of co-crystallized oligonucleotide. Amino acids of FANCM contacting DNA are shown in black (backbone-backbone contacts), red (sidechain-backbone contacts), blue (backbone-to-base contacts) and green (sidechain-base contacts).

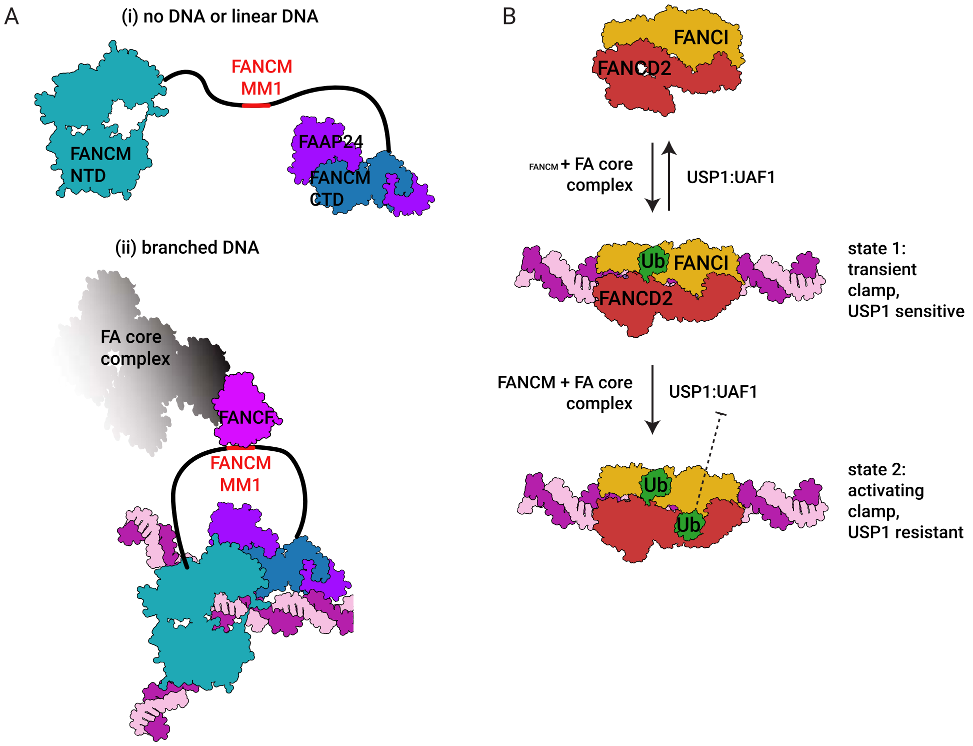

Figure EV5. Models based on data generated in this manuscript aligned to existing literature.

(**A**) FANCM also exists in at least 2 states: (i) a free state where the N-terminus and C-terminus are not associated, and the protein is not activating the ubiquitination dependent pathway and (ii) a DNA bound state where the N- and C-terminus come together which activates the complex to monoubiquitinate FANCD2, but drive the enzyme to sequentially and rapidly ubiquitinate FANCI. In this way, FANCM drives the quick progression of the transient ID2 clamp shown in (**A**) to the activating clamp required for DNA repair signaling. The motor of FANCM also becomes activated, to stabilize the fork structure, required for the downstream repair steps. (**B**) Dual monoubiquitination converts FANCD2:FANCI from a transient to stable clamp on DNA.

 © The Author(s)
